# Identification of FISH biomarkers to detect chromosome abnormalities associated with prostate adenocarcinoma in tumour and field effect environment

**DOI:** 10.1186/1471-2407-14-129

**Published:** 2014-02-25

**Authors:** Ying Zhang, Thomas Perez, Beth Blondin, Jing Du, Ping Liu, Diana Escarzaga, John S Coon, Larry E Morrison, Katerina Pestova

**Affiliations:** 1 Abbott Molecular, Inc, 1300 East Touhy Avenue, Des Plaines, IL 60018, USA; 2 Department of Pathology, Rush University Medical Center, 1750 West Harrison Street, Chicago, IL 60612, USA

**Keywords:** Prostate cancer, Genomic abnormalities, Diagnosis, Field effect, Fish, Fluorescence in situ hybridisation

## Abstract

**Background:**

To reduce sampling error associated with cancer detection in prostate needle biopsies, we explored the possibility of using fluorescence in situ hybridisation (FISH) to detect chromosomal abnormalities in the histologically benign prostate tissue from patients with adenocarcinoma of prostate.

**Methods:**

Tumour specimens from 33 radical prostatectomy (RP) cases, histologically benign tissue from 17 of the 33 RP cases, and 26 benign prostatic hyperplasia (BPH) control cases were evaluated with Locus Specific Identifier (LSI) probes *MYC* (8q24), *LPL* (8p21.22), and *PTEN* (10q23), as well as with centromere enumerator probes CEP8, CEP10, and CEP7. A distribution of FISH signals in the tumour and histologically benign adjacent tissue was compared to that in BPH specimens using receiver operating characteristic curve analysis.

**Results:**

The combination of *MYC* gain, CEP8 Abnormal, *PTEN* loss or chromosome 7 aneusomy was positive in the tumour area of all of the 33 specimens from patients with adenocarcinomas, and in 88% of adjacent histologically benign regions (15 out of 17) but in only 15% (4 out of 26) of the benign prostatic hyperplasia control specimens.

**Conclusions:**

A panel of FISH markers may allow detection of genomic abnormalities that associate with adenocarcinoma in the field adjacent to and surrounding the tumour, and thus could potentially indicate the presence of cancer in the specimen even if the cancer focus itself was missed by biopsy and histology review.

## Background

Prostate carcinoma is the most common type of cancer in men in the United States, with an estimated 241,740 new cases in 2012 and 28,170 deaths [1]. It is the second leading cause of cancer death in US men after lung cancer. The absence of reliable diagnostic markers that enable early and accurate detection of carcinomas when they are confined to the prostate is a fundamental problem in the management of prostate cancer. The leading early detection and diagnostic approach employs a combination of DRE (digital rectal examination) and measurement of serum PSA (prostate-specific antigen) followed by a prostate biopsy. However, this approach has major limitations. Out of the approximately 1.2 million patients who undergo prostate biopsy each year in the US, 70% to 80% receive negative results [2]. These patients cannot be completely reassured, however, because a cancer might have been missed by sampling error due to the focal nature of Prostate cancer (CaP) [3,4]. Therefore, each year about 840,000 to 960,000 men undergo repeat biopsies because of consistently elevated PSA levels [4]. An additional challenge in prostate cancer diagnosis is that prostate cancer is a multi-focal disease, with 67% to 96% of radical prostatectomy specimens containing more than one focus of disease [5-7]. Studies have shown that the use of more biopsy cores may improve accuracy of diagnosis, reducing the sampling effect [8]. Currently, a 12-core scheme is recommended as a reasonable biopsy approach, providing an acceptable sampling of the prostate gland [9], however, collection of a higher number of biopsy cores is also being considered [2]. Due to the potential comorbidity associated with collection of the high number of biopsy cores, utilisation of molecular assays to improve the diagnostic accuracy would be beneficial. Combined with conventional histopathological assessment, predictive biomarkers that indicate the high likelihood of the presence of malignancy on a biopsy specimen without clearly malignant histology could provide clinicians with guidance for stratifying individuals into those who need repeat biopsy or intensive follow-up and those who do not.

The concept of “field cancerisation” or “field effect” was first proposed by Slaughter et al. in 1953 when observing histological features of oral cancers [10] to describe the presence of histologically abnormal tissue surrounding primary cancerous lesions. It was considered to cause the occurrence of multifocal tumours and cancer recurrence. Due to the tremendous progress in molecular biology and biotechnology, the definition of field effect has been extended to the molecular abnormalities in tissues that appear histologically benign, as defined by Höckel and Dornhöfer [11]: “the monoclonal or multiclonal displacement of normal epithelium by a genetically altered but microscopically undistinguishable homologue.” Since then, the presence of the field effect has been reported in various tumour types, including carcinoma of the head and neck, lung, colon and rectum, breast, stomach, prostate, and urinary bladder [12,13].

Prostate cancer is multifocal disease, and field effect may play a fundamental role in the development of multifocal lesions. A recent review by Trujillo KA et.al [14] summarized that field cancerisation of prostate can occur at the levels of genetic, epigenetic, and biochemical aberrations in structurally intact cells in histologically normal tissues adjacent to cancerous lesions. Prostate cancer biomarkers of field cancerisation have been studied by several groups using different strategies, including nuclear morphology, DNA methylation, Mitochondrial DNA changes, mRNA profiling, protein expression and genomic DNA changes [14]. Genomic, epigenetic, and biochemical alterations observed outside the histologically visible tumour margins could result from pre-existing fields of precursor cells in which cancer develops; alternatively, the tumour could have an effect on the surrounding tissue, or the observed abnormalities could reflect both of the above effects [15]. The question whether the field of molecular alterations is exclusively of precursor nature, or whether it is induced by the tumour, is still being discussed in the literature [14]. However, irrespective of their origin, the markers of field cancerisation are associated with the cancer, and could indicate the presence of cancer in the specimen if detected in the tumour-adjacent histologically benign tissue.

Although Florescence In Situ Hybridization technique (FISH) represents a molecular technique that allows the detection of numerical and structural genomic abnormalities in interphase cell nuclei in tissue sections or cytological specimens such as deletion, amplification, and translocation of various genomic regions in many types of cancer [16,17], it has not been widely used in the studies on field cancerisation. Multiple chromosomal alterations have been reported in CaP [18-20], including chromosome aneusomy, gain of the 8q24 (*MYC*) locus, and loss of 8p21-22 (*LPL*) [21], and 10q23 (*PTEN*), among others [22]. In our initial feasibility study, we evaluated aberrations in multiple genomic loci involved in tumorigenesis by FISH on a set of FFPE prostate adenocarcinoma specimens, and selected a group of probes that detected cytogenetic abnormalities in these tumours. The selected probes included *LPL, MYC, PTEN*, CEP7, CEP 8, and CEP 10. In this study, using FISH technique, we assessed whether these biomarkers could detect chromosomal abnormalities that are present in the histologically benign region adjacent to frank carcinoma.

## Methods

### FISH probes

A total of 6 probes including 3 centromeric probes (CEP®) and 3 locus-specific identifiers (LSI®) were used. CEP probes included CEP7 (SpectrumAqua™), CEP8 (SpectrumAqua), and CEP10 (SpectrumGreen™). LSI probes were *PTEN* (SpectrumOrange™), *MYC* 8q24 (SpectrumGreen), and *LPL* 8p21-22 (SpectrumOrange). All probes were obtained from Abbott Molecular, Inc. (Des Plaines, IL).

### Histological specimen collection

Thirty-three archived RP cases from patients with prostate adenocarcinoma and 26 control Benign Prostatic Hyperplasia (BPH) cases were provided by Rush University Medical Center (Approved IRB L06052503 waived the requirement for informed consent). Multiple tissue blocks were prepared from each of the RP cases. For each specimen, 5 μm tissue sections were cut and placed on positively charged microscope slides. The blocks were characterised by staining one out of 10 serial sections through the block with haematoxylin and eosin (H&E) followed by examination by an expert pathologist. For all 33 cases, at least one block containing adenocarcinoma was identified for this study and designated as “tumour”. Region(s) with histopathological features of adenocarcinoma were marked by the pathologist on the H&E slides of the tumour specimens. Twenty-five of the 33 adenocarcinoma cases were determined to have a Gleason score of 5–7, 6 cases had a Gleason score of >7, and 2 had a Gleason score of 2–4. For 17 out of 33 adenocarcinoma cases used in this study, a second block was identified that contained no recognisable histological features of adenocarcinoma or prostatic intraepithelial neoplasia (PIN) and designated as “histologically benign.” It was estimated that histologically benign tissue was spatially separated from the tumour margin on average by approximately 1 cm. The H&E images of the 17 histologically benign slides used in the study are provided in the Additional file 1. For the BPH cases, FFPE blocks of TURP specimens were utilized. For each TURP specimen, 5 μm tissue sections were cut and placed on positively charged microscope slides. The blocks were characterised by staining one out of 10 serial sections through the block with haematoxylin and eosin (H&E) followed by examination by an expert pathologist to confirm that no histological features of adenocarcinoma or prostatic intraepithelial neoplasia (PIN) are present.

The specimen slides used for the FISH assay procedure were within 10 serial sections of the respective H&E-stained slide to assure minimal separation of the areas examined by FISH from the areas evaluated by histopathology.

### Histological sample pre-treatment and hybridisation

Formalin fixed paraffin embedded (FFPE) histological specimen slides were baked at 56°C for 2–24 hours, then were treated three times in Hemo-De (Scientific Safety Solvents) for 5 minutes each at room temperature followed by two 1-minute rinses in 100% ethanol at room temperature. Slides were incubated in pre-treatment solution (1× SSC, pH7.0) at 80°C for 35 minutes, rinsed for 3 minutes in deionized water, incubated 20–22 minutes in 0.15% pepsin in 0.1 N HCl solution at 37°C, and rinsed again for 3 minutes in deionized water. Slides then were dehydrated for 1 minute each in 70%, 85%, and 100% ethanol and air-dried. Two sets of probe hybridisation mix were made: Probe mix 1 included CEP8 (SpectrumAqua), *MYC* 8q24 (SpectrumGreen), and *LPL* 8p21-22 (SpectrumOrange); Probe mix 2 consisted of CEP7 (SpectrumAqua), CEP10 (SpectrumGreen), and *PTEN* (SpectrumOrange). Ten microliters of either probe hybridisation mix containing blocking DNAs and LSI/WCP Hybridisation Buffer (Abbott Molecular, Inc., Des Plaines, IL) were added to a specimen, and a coverslip was applied with rubber cement sealed around. Slides and probes were codenatured for 5 minutes at 73°C and hybridized for 16–24 hours at 37°C on a ThermoBrite® hybridisation platform (Abbott Molecular, Inc.). Following hybridisation, coverslips were removed by soaking the slides in 2× SSC/0.3% NP-40 for 2–5 minutes, and immediately slides were washed in 2× SSC/0.3% NP-40 at 73°C for 2 minutes and subsequently in 1× SSC solution, PH ~ 7.0 for 1 minute at room temperature. The slides were then allowed to dry in the dark. Ten microliters of 4',6-diamidino-2-phenylindole counterstain/antifade solution (DAPI I, Abbott Molecular, Des Plaines, IL) was added to the specimen, and a coverslip was placed on the slide for microscopy.

### FISH signal evaluation

The specimens were analysed under a fluorescence microscope using single bandpass filters (Abbott Molecular, Des Plaines, IL) specific for DAPI, SpectrumOrange, SpectrumGreen, and SpectrumAqua. The number of FISH signals for each probe was recorded in a minimum of 50 consecutive non-overlapped, intact interphase nuclei in areas of interest, which were identified by DAPI staining of nuclei with reference to the corresponding H&E-stained tissue. Tumour areas (tumour regions of interest, ROI) scribed by the pathologist were evaluated on the 33 RP specimen slides. For the 17 slides with histologically benign tissue adjacent to tumour, representative areas were evaluated (histologically benign regions of interest, ROI). Similarly, representative areas were evaluated on BPH specimen slides.

### Statistical analysis

For each specimen, 50–100 cells were enumerated with respect to the number of fluorescent signals of each probe. The following FISH abnormality parameters were calculated for each probe:

• %Gain, percent cells with > 2 signals

• %Loss, percent cells with < 2 signals

• %Abnormal, percent cells with > 2 or < 2 signals

• For the two probe ratios (probe A/probe B), %Gain is the percentage of cells with A/B ratio >1, and %Loss is the percentage of cells with A/B ratio < 1.

In order to screen for FISH probes potentially important for disease detection, the FISH parameters described above were compared between different specimen groups (tumour ROI vs. BPH, and histologically benign ROI vs. BPH) using a two-sample *t*-test. FISH parameters with significant p-values (p-value < 0.05) from the *t*-test were selected for further examination.

After prioritizing potential FISH probes, the receiver operating characteristic (ROC) method [23] was used to select optimal FISH probe combinations, as well as the optimal cut-off value for individual FISH probes. The ROC curve is a plot of sensitivity versus 1-specificity or false positive rate (FPR). In our study, each point on the ROC curve represents a sensitivity/specificity pair corresponding to a particular cut-off (for single FISH parameter) or a combination of cut-offs (for FISH parameter combinations). From the ROC curves, the distance from ideal (DFI) and the area under the curve (AUC) were calculated.

DFI is defined as $\sqrt{{\left(1-\mathrm{sensitivity}\right)}^{2}+{\left(1-\mathrm{specificity}\right)}^{2}}$. DFI represents the minimum distance from the ROC curve to the value of a sensitivity of 1 and a false positive rate (1-specificity) of 0. The DFI ranges from 0 to 1, with 0 being the ideal. Determination of the “optimal" cut-off value is always a trade-off between sensitivity and specificity. Ideally, the “optimal" cut-off value provides both the highest sensitivity and the highest specificity, easily located on the ROC curve by finding the point with minimum DFI. For determination of optimal cut-off value, minimal DFI was used as the selection criterion. The parameters with the determined cut-off below the level of truncation for the FISH signals in FFPE tissue specimens were not included in the further analysis. For determination of optimal FISH probe combination, AUC was used as the selection criterion. Statistically, the best FISH probe combination is the one with largest AUC values. For the selection of the final, optimized probe set, scientific judgment was applied in combination with the statistical analysis.

All analyses were performed using SAS version 9.2 (SAS Institute Inc., Cary, NC, USA.) on a UNIX operating system by Abbott Molecular Biostatistics and Data Management Group.

## Results

### Detection of cytogenetic abnormalities by FISH in RP specimens

In an initial feasibility study, we tested 16 RP specimens from patients with adenocarcinoma of the prostate. Nine out of the 16 specimens had a second section available with only histologically benign tissue. Slides from 11 BPH cases were used as controls. The probes included LSI *MYC* (8q24), *LPL* (8p21.22), *PTEN* (10q23), and p16 (9p21), centromere probes CEP8, CEP10, CEP7, CEP3, and CEP17, and *TMPRSS2* break-apart. In the study, we observed that tumour ROIs in radical prostatectomy specimens bore chromosomal abnormalities, including *MYC* amplification/gain, *LPL* and *PTEN* loss, *TMPRSS2* rearrangement, as well as general aneuploidy. Importantly, we also observed chromosomal abnormalities in some of the 9 histologically benign slides from tumour patients. The results demonstrated the feasibility of using a FISH assay to detect chromosome abnormalities that are specific to specimens from adenocarcinoma patients. Based on these initial results, we selected six probes that detected chromosomal copy number abnormalities in most RP specimens, both within tumour regions and extending beyond histologically evident tumour. The six probes included *MYC, LPL,* CEP8, *PTEN*, CEP10, and CEP7.

We expanded the initial specimen set to the total of 33 RP specimens from patients with adenocarcinoma of prostate and 26 BPH control specimens for the interrogation with the six selected FISH probes. For 17 out of 33 adenocarcinoma cases, in addition to the tumour region, we also evaluated tumour-adjacent histologically benign tissue from the same specimen, using a separate FFPE block that contained no histological features of adenocarcinoma or prostatic intraepithelial neoplasia (PIN) upon histopathological examination.

**Figure 1:**
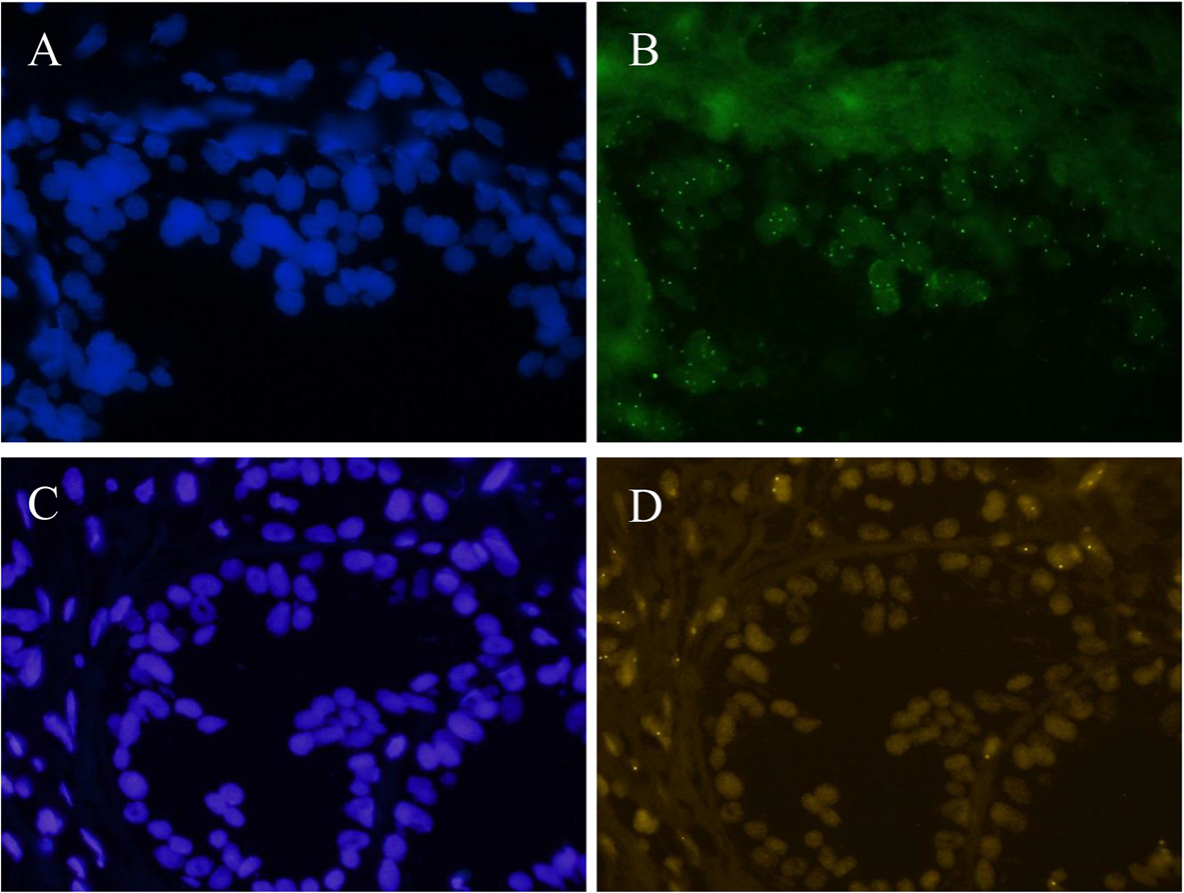
**Images of abnormal FISH signals. A** and **B**: The images of MYC SpectrumGreen copy number gain with DAPI staining **(A)** showing the nuclei morphology, and MYC staining **(B)** displaying gain of copy numbers. **C** and **D**: the images of PTEN loss with DAPI staining **(C)** of the tumour ROI, and PTEN FISH hybridisation showing deletion of PTEN in the prostate gland **(D)**.

Chromosomal abnormalities of *MYC, LPL* and *PTEN*, and aneusomy (as measured by copy number changes of the chromosome-specific CEP probes) were observed in tumour ROIs of the radical prostatectomy specimens. Figure 1 shows images representing the copy number gain of MYC (Figure 1A and B) and the loss of PTEN (Figure 1C and D). Images were recorded within the tumour ROI, which could also be recognized by the characteristic pattern of nuclei under DAPI staining (Figure 1A and C). MYC signal was clearly gained (Figure 1B, displaying 3 or 4 signals per cell in this specimen), while PTEN signal was lost (Figure 1D), showing either zero or one copies in the majority of cells within the ROI.

In this study, FISH analysis was performed on histological specimens. In contrast to cytology, FISH on FFPE tissue specimens presents artefacts related to the nuclear truncation. In our study, FISH analysis of individual signal counts showed a loss up to 10% of signals, with average counts of FISH probe signals per cell of 1.80-1.84 in both test (RP) and control (BPH) tissue specimens. Therefore, although truncation effects were evident, the level of truncation did not appear to differ between test and control cases. To further control for artefacts of nuclear truncation, the cut-offs for FISH positivity, especially for deletion probes, were chosen above the level of truncation, as presented in the section Data Analysis of Probe Performance below.

Sixteen parameters derived from genomic copy numbers detected by FISH were evaluated by the *t*-test comparing tumour ROI and BPH. These parameters were CEP10%Abnormal, CEP10%Gain, CEP7%Abnormal, CEP7%Gain, CEP8%Abnormal, CEP8%Gain, CEP8%Loss, *MYC*%Gain, *LPL*%Abnormal, *LPL*%Loss, *PTEN*%Loss, *PTEN*/CEP10%Loss, CEP7/CEP10%Gain, *LPL*/CEP8%Loss, *MYC*/CEP8%Gain and *MYC*/*LPL*%Gain. Results from *t*-test analyses demonstrated that for all of the 16 FISH parameters, mean values were statistically different between tumour and BPH groups (Additional file 2: Table S2a). Interestingly, chromosomal abnormalities were observed not only in the tumour ROIs, but also on slides with the histologically benign tissue approximately 1 cm away from the tumour margin of the RP adenocarcinoma specimens (benign ROI).

**Figure 2:**
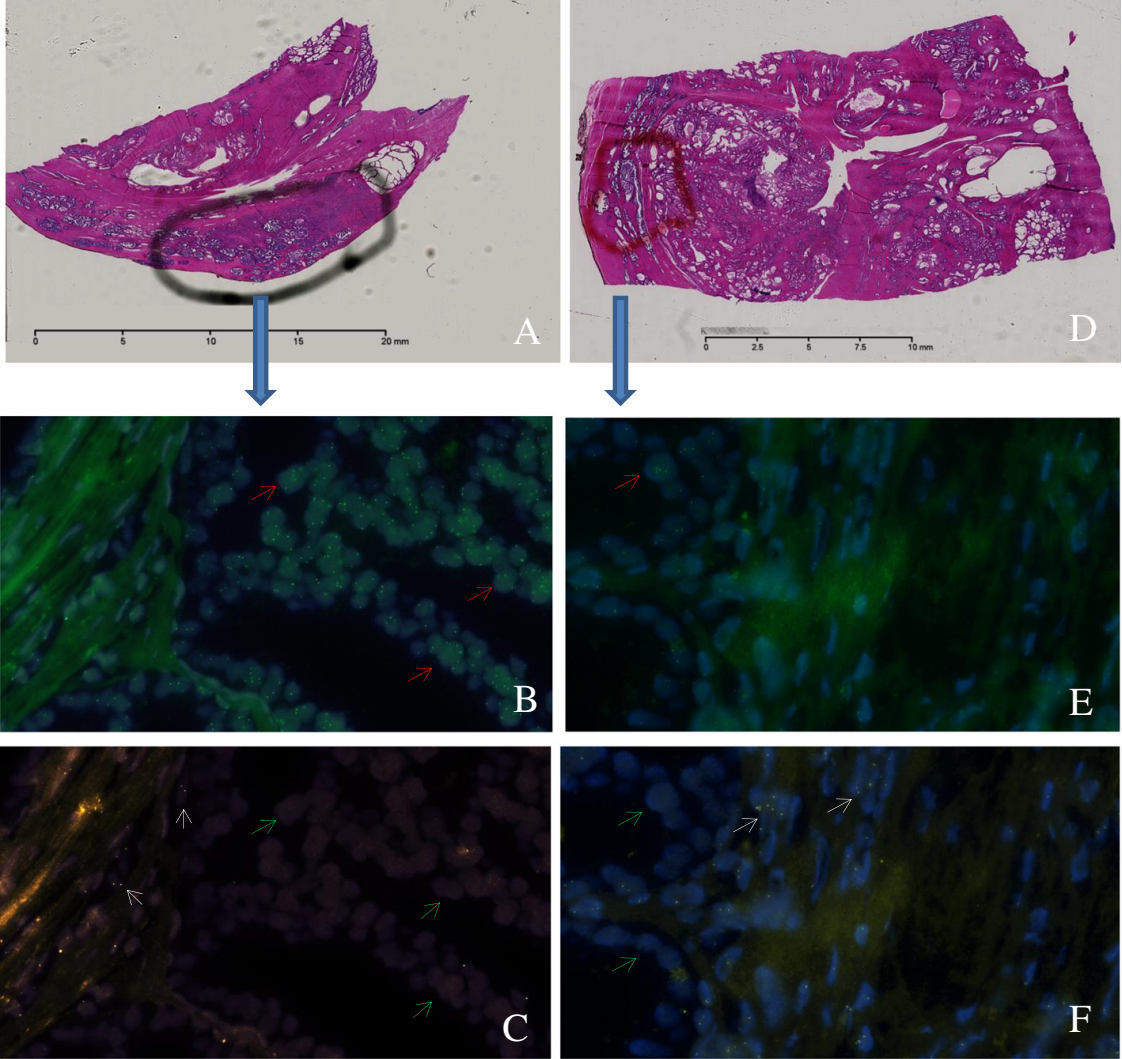
**Representative images of FISH and H&E staining from tumour and histologically benign areas of the same case. A**: an H&E image of a tumour section (from Case 01, tumour block) with the area of tumour circled; **B**: MYC amplification (green signals indicated by the red arrow) and DAPI nuclear staining (blue) in a representative field of view of the tumour section; **C**: PTEN deletion indicated by green arrows and DAPI nuclear staining (blue) in a representative field of view of the tumour section (gold PTEN signals are visible only in stroma cells); **D**: an H&E image of the histologically benign section (from Case 01, histologically benign block) with the area that has abnormal FISH signals circled; **E**: MYC amplification (green signals indicated by the red arrow) and DAPI nuclear staining (blue) in a representative field of view of the histologically benign section; **F**: PTEN deletion indicated by green arrows and DAPI nuclear staining (blue) in a representative field of view of the histologically benign section (gold PTEN signals are visible only in stroma cells, indicated by white arrows).

Representative images demonstrating FISH and H&E staining from a tumour section (Figure 2A, 2B, and 2C) and a histologically benign section (Figure 2D, 2E, and 2F) of the same case are shown in Figure 2. Figure 2A presents an H&E image of a tumour section (from Case 01, tumour block) with the area of the tumour circled. Figure 2D shows an H&E image of the histologically benign section (from Case 01, histologically benign block) with the area that had abnormal FISH signals circled. Figure 2B and 2C show abnormal FISH in a representative field of view of the tumour section. Figure 2E and 2F show abnormal FISH from a representative field of view of the histologically benign section. Figure 2B and 2E show MYC amplification (green signals indicated by the red arrow) and DAPI nuclear staining (blue), while Figure 2C and 2F show PTEN deletion indicated by green arrows (gold PTEN signals are visible only in stroma cells, indicated by white arrows) and DAPI nuclear staining (blue).

To confirm that the histologically benign areas selected for the study were indeed devoid of tumour features, we utilized a second, independent pathologist to review the corresponding H&E images. Upon the in-depth review of the suspected area, all but one specimen were deemed to be histologically normal, since no features of prostate adenocarcinoma, or PIN were observed in these regions. In one apparently benign specimen (sample number 33 block B), a very close, detailed inspection by the independent pathologist revealed possible minute tumour foci, however, the pathologist was not able to conclusively classify the observed cells as tumour without a suggested confirmation by other methods (such as IHC), highlighting the challenges in histopathological assessment of prostate tissue specimens. Therefore, we confirmed that FISH detected cytogenetic abnormalities in the regions of prostate that would not have been identified as tumour on histopathology review.

Results from t-tests comparing histologically benign ROI and BPH controls, showed that for 10 out of the 16 FISH parameters (Additional file 2: Table S2b), mean values were statistically different between the comparison groups. These 10 FISH parameters, derived from copy numbers of MYC, LPL, CEP8, PTEN, CEP10 and CEP7, were CEP10%Abnormal, CEP10%Gain, CEP7%Abnormal, CEP7%Gain, CEP8%Abnormal, CEP8%Gain, MYC%Gain, LPL% Abnormal, PTEN%Loss, PTEN/CEP10%Loss.

### Analysis of probe performance and selection of optimal probe combinations

The receiver operating characteristic (ROC) analysis method was applied to the selected ten single FISH parameters. AUC values from the ROC analysis comparing histologically benign ROIs from adenocarcinoma RP specimens with BPH controls were used to assess the selected FISH parameters in respect to their ability to distinguished adenocarcinoma specimens from the BPH controls based on the presence of the genomic abnormalities extending beyond the tumour margin. Table 1 summarizes the sensitivity and specificity of the selected FISH parameters at the optimal cut-off for each parameter. In this analysis, the cut-off was determined based on the shortest Distance From Ideal (DFI) as described in Methods. All of the selected parameters were also highly abnormal in tumour area of adenocarcinoma RP specimens, as compared to BPH control.

**Table 1:** ROC analysis (sensitivity, specificity, and the AUC) comparing ten selected FISH probe parameters

**FISH probe parameters**	**Tumour - BPH**			**Histologically benign - BPH**		
	**Sensitivity**	**Specificity**	**AUC**	**Sensitivity**	**Specificity**	**AUC**
CEP8%Gain	79.40%	76.90%	0.853	82.40%	76.90%	0.871
*MYC*%Gain	91.20%	76.90%	0.908	82.40%	76.90%	0.846
CEP7%Abnormal	88.20%	80.80%	0.896	76.50%	80.80%	0.845
*PTEN*%Loss	58.80%	80.80%	0.726	70.60%	80.80%	0.827
*PTEN*/CEP10%Loss	82.40%	80.80%	0.881	64.70%	80.80%	0.788
CEP10%Gain	94.10%	84.60%	0.93	76.50%	84.60%	0.786
CEP10%Abnormal	79.40%	80.80%	0.865	76.50%	73.10%	0.775
CEP7%Gain	85.30%	84.60%	0.886	70.60%	76.90%	0.77
CEP8%Abnormal	94.10%	88.50%	0.966	64.70%	76.90%	0.752
*LPL*%Abnormal	79.40%	92.30%	0.945	64.70%	76.90%	0.736

The analyses of tumour ROI vs. BPH, and benign ROI vs. BPH performed independently. Sensitivity and specificity calculated at the optimal cut-off in each of the two analyses for each parameter.

The selected single FISH probe parameters listed in Table 1 were then grouped in all possible 4-probe combinations, and the ROC method was used now to identify the optimal probe combinations based on their AUC values and clinical consideration in distinguishing histologically benign ROI specimens vs. BPH. Table 2 shows the selected 4 probe combinations identified in this analysis.

**Table 2:** ROC analysis of the selected four 4-probe combinations from the 10 single FISH probe parameters

**Probe combination**	**Probe parameters**	**Cut-off 1**	**Cut-off 2**	**Cut-off 3**	**Cut-off 4**	**Sensitivity**	**Specificity**	**AUC**
Probe combination 1	*PTEN*%loss, CEP7%abnormal, *MYC*%gain, CEP8%abnormal	33 PTEN% loss	28 CEP7% abnormal	35 MYC% gain	34 CEP8% abnormal	88.20%	84.60%	0.917
Probe combination 2	*PTEN*/CEP10%loss, CEP7%abnormal, *MYC*%gain	35 *PTEN*/CEP10% loss	25 CEP7% abnormal	35 *MYC*% gain	NA	82.40%	80.80%	0.911
Probe combination 3	*PTEN*/CEP10%loss, CEP7%abnormal, CEP8%abnormal	35 PTEN/CEP10% loss	25 CEP7% abnormal	34 CEP8% abnormal	NA	88.20%	80.80%	0.938
Probe combination 4	*PTEN*/CEP10%loss, *MYC*%gain, CEP8gain	35 PTEN/CEP10% loss	5 MYC% gain	35 CEP8% gain	NA	64.70%	88.50%	0.834

Cut-off 1, cut-off 2, cut-off 3, and cut-off 4 are the optimal probe cut-offs (providing lowest DFI), respectively.

As evident from Table 2, probe combination 3 had the highest AUC value of 0.938, while probe combination 1 had the second highest AUC value (0.917) and afforded the lowest DFI (combined highest sensitivity and specificity) at the indicated cut-offs. Since the achievable combined sensitivity and specificity were noticeably higher for probe combination 1, and since this probe combination also contained probes to two important tumour-related loci, probe combination 1 was selected over probe combination 3 despite the somewhat higher AUC achieved with probe combination 3. The optimal cut-off values chosen for the four individual FISH probe parameters in probe combination 1 are *PTEN*%loss > 33, CEP7%Abnormal > 28, *MYC*%gain > 35, and CEP8%Abnormal > 34. Using these cut-offs, probe combination 1 yielded a sensitivity of 88.2% and a specificity of 84.6% for histologically benign ROI vs. BPH (AUC = 0.917), while the sensitivity and specificity for tumour ROI vs. BPH were 100% and 84.6%, respectively, with the AUC = 0.960.

**Figure 3:**
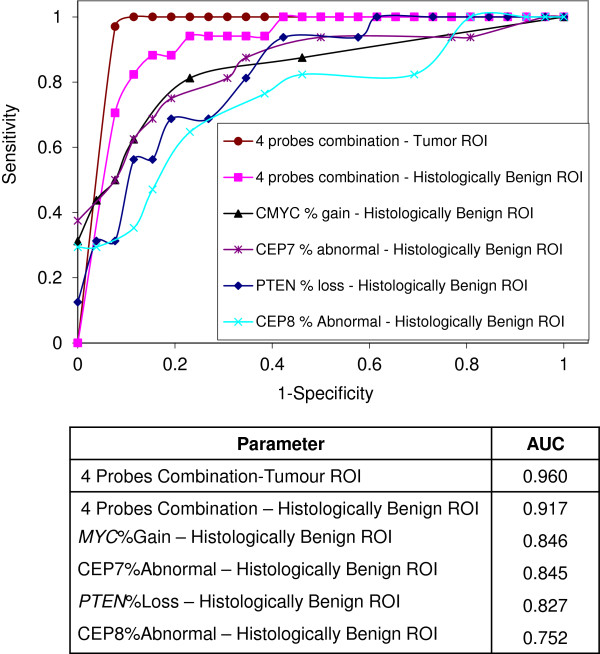
**ROC curve.** ROC plot for individual FISH probe parameters (PTEN%loss, CEP7%abnorm, MYC%gain, CEP8%abnormal) and the 4-probe combination. Data were calculated from the FISH evaluation of the 17 benign ROI (histologically benign regions surrounding the tumour) and 26 BPH specimens. The ROC plot for the 4-probe combination of the 33 tumour ROI and 26 BPH specimens are also shown here. The AUC of the ROC curves are shown in the table.

ROC curves for the selected 4- probe combination, and the corresponding 4 single FISH probe parameters, including *PTEN*%loss, CEP7%Abnormal, *MYC*%gain, CEP8%Abnormal, are plotted in Figure 3. The curves labelled ‘benign ROI’ were obtained from the FISH evaluation comparing the 17 histologically Benign ROI to the 26 BPH specimens. The curve labelled ‘tumour ROI’ was obtained from the evaluation comparing the 33 tumour ROI to the 26 BPH. The corresponding AUC values for each of the plotted ROC curves are listed in the table under the ROC plot.

## Discussion

Each year, millions of men are referred for prostate biopsies due to abnormal DRE or elevated serum PSA. Prostate biopsies are not only unpleasant, but also carry risks to the patient, and are expensive. Moreover, false-negative rates for initial prostate biopsies (particularly for sextant biopsies) are routinely reported to be between 10–25%, and repeat biopsies are essential components of prostate cancer detection [24]. Therefore, reliable diagnostic markers that enable early and accurate detection of prostate tumours when they are confined to the prostate are essential.

In this study, we tested *LPL*, *MYC*, *PTEN*, CEP 7, CEP 8, and CEP 10 FISH probes, based on the published roles of these genes, and on our initial FISH study on FFPE prostate cancer and BPH specimens. Sixteen parameters were derived from the number of FISH signals at the above loci, and compared between prostate adenocarcinoma tumour tissue (tumour ROI) and BPH samples by the *t*-test. In this analysis, all 16 FISH parameters demonstrated a significant difference between the comparison groups, supporting the role of these genes in prostate cancer. In addition to the tumour ROIs, we found FISH abnormalities in histologically benign tissue separated from the tumour margin on average by approximately 1 cm (histologically benign ROI).

The pattern of FISH abnormalities in the histologically benign ROIs was similar to that found in the corresponding tumour (Additional file 3). The observed cytogenetic abnormalities appeared to be reflective of genetic changes in the cancer cells of the associated tumour, indicating the possibility that a field cancerisation effect may be manifested in prostate cancer at the cytogenetic level as a field of molecular alterations in adjacent, histologically benign areas surrounding the tumour. Genomic alterations observed outside the histologically visible tumour margins could result from pre-existing fields of precursor cells in which cancer develops; alternatively, the tumour could have an effect on the surrounding tissue, or the observed abnormalities could reflect both of the above abnormalities [15]. We also cannot rule out that the cells in which genomic abnormalities were detected represent events related to the metastatic tumour spread, such as undetected micro-metastases. However, although FISH does not have an ability to establish whether a cell under interrogation is a tumour cell, a precursor cancer cell or a benign cell, cytogenetic abnormalities detected by FISH in our study were similar to those in the associated tumour.

Not all chromosomal loci were detectable in the field surrounding the tumour at the same level. Out of the initial sixteen FISH parameters chosen for evaluation, 10 were identified that detected FISH abnormalities in histologically benign ROIs of RP adenocarcinoma specimens, specifically CEP10%Abnormal, CEP10%Gain, CEP7%Abnormal, CEP7%Gain, CEP8%Abnormal, CEP8%Gain, *MYC*%Gain, *LPL*%Abnormal, *PTEN*%Loss, *PTEN*/CEP10%Loss.

By combining the individual FISH parameters, we identified a probe combination (*PTEN*, *MYC*, CEP7 and CEP8) that was superior in performance to that of individual probes (Table 2 and Figure 3). In the specimen set used in this study, with the optimal cut-offs for each FISH parameter selected using the ROC method, the sensitivity of the 4-probe combination was 88.2% and the specificity was 84.6% for discriminating prostate adenocarcinoma from BPH specimens based on cytogenetic abnormalities found in histologically benign regions surrounding the tumour. Using the same cut-offs for each FISH parameter, a sensitivity of 100% and a specificity of 84.6% were achieved for discriminating prostate adenocarcinoma from BPH specimens based on cytogenetic abnormalities found within the tumour. These findings support the field cancerisation effect of prostate cancer reported in several studies, including some of the recent work of methylation changes [25], Telomere attrition [26], Mitochondrial DNA changes [27], and Gene expression changes [28,29]. Significantly, evidence for such malignancy-associated changes has been presented in other organs such as the cervix, bladder and breast [11].

The results of our feasibility study of radical prostatectomy suggest that multi-colour FISH may find utility in detecting cytogenetic abnormalities associated with adenocarcinoma of prostate in the field around the tumour, and therefore could potentially aid in assessing negative biopsies of patients with suspected cancer. The hypothesis that FISH could be used to aid the detection of adenocarcinoma by assessing the tissue surrounding the tumour will be validated in the next phase of the investigation using prostate needle biopsy specimens.

## Conclusions

In this study of radical prostatectomy specimens, cytogenetic abnormalities were observed by FISH within regions of prostate adenocarcinoma, as well as within regions of benign histology extending beyond histologically evident tumour margin, indicating a field cancerisation effect in prostate cancer. Detection of field cancerisation by FISH may prove to have a utility in the evaluation of histology-negative biopsies from patients suspected of having prostate cancer, and therefore could aid histopathological evaluation by providing an indication of possible presence of malignancy. Although preliminary, the findings of a FISH panel with sensitivity >85% in histologically benign regions away from tumour and specificity ~85% in BPH, provide encouragement to pursue the utility of these cytogenetic markers further. Validation in a larger cohort of patients with both positive and negative biopsies is envisioned to confirm our findings.

## Abbreviations

AUC: Area under the curve; BPH: Benign prostatic hyperplasia; CaP: Prostate cancer; CEP: Centromeric probes; DRE: Digital rectal examination; FFPE: Formalin fixed paraffin embedded; FISH: Fluorescence in situ hybridisation; LSI: Locus specific identifier; PSA: Prostate-specific antigen; ROC: Receiving operating characteristic; RP: Radical prostatectomy; ROI: Region of interest.

## Competing interests

Authors YZ, KP, LM have filed a pending patent application relating to the subject matter of this article, which patent application has been assigned to Abbott Molecular Inc.

## Authors’ contributions

YZ analysed the histological samples, performed the statistical analysis, and drafted the manuscript. TP analysed the histological samples. BB analysed the histological samples. JD performed the statistical analysis and helped to draft the statistical section. PL performed the statistical analysis and helped to draft the statistical section. DE assisted in the appropriate specimen selection and sectioned the slides. JC provided clinical opinion, selected and provided appropriate specimens and helped conceive the study, and scribed the tumour region on the H&E slides. LM conceived the study and the study design, provided guidance on data analysis, KP coordinated the study and its design, analysed the histological samples, assisted in selection of probes, performed the statistical analysis, and helped draft the manuscript. All authors read and approved the final manuscript.

## Pre-publication history

The pre-publication history for this paper can be accessed here:

http://www.biomedcentral.com/1471-2407/14/129/prepub

## Supplementary Material

Additional file 1 **H&E Images of the 5-μm sections of the 17 histologically benign specimens from the corresponding radical prostatectomy adenocarcinoma cases.** For each case, the specimen slide used for the FISH analysis was within 10 serial sections of the H&E stained slide. Click here for file

Additional file 2 ***t*-test analyses. Table S2a.***t*-test of tumour ROI vs BPH. **Table S2b.***t*-test of histologically benign ROI vs BPH. Click here for file

Additional file 3 The pattern of FISH abnormalities presents in the 33 tumour ROIs and the 17 available corresponding histologically benign ROIs. Click here for file
