# Supplementary material for: Identification of FISH biomarkers to detect chromosome abnormalities associated with prostate adenocarcinoma in tumour and field effect environment
Source: BMC Cancer. 2014 Feb 25;14:129. doi: 10.1186/1471-2407-14-129 (PMC4016502; doi:10.1186/1471-2407-14-129)

**Additional file 1.**

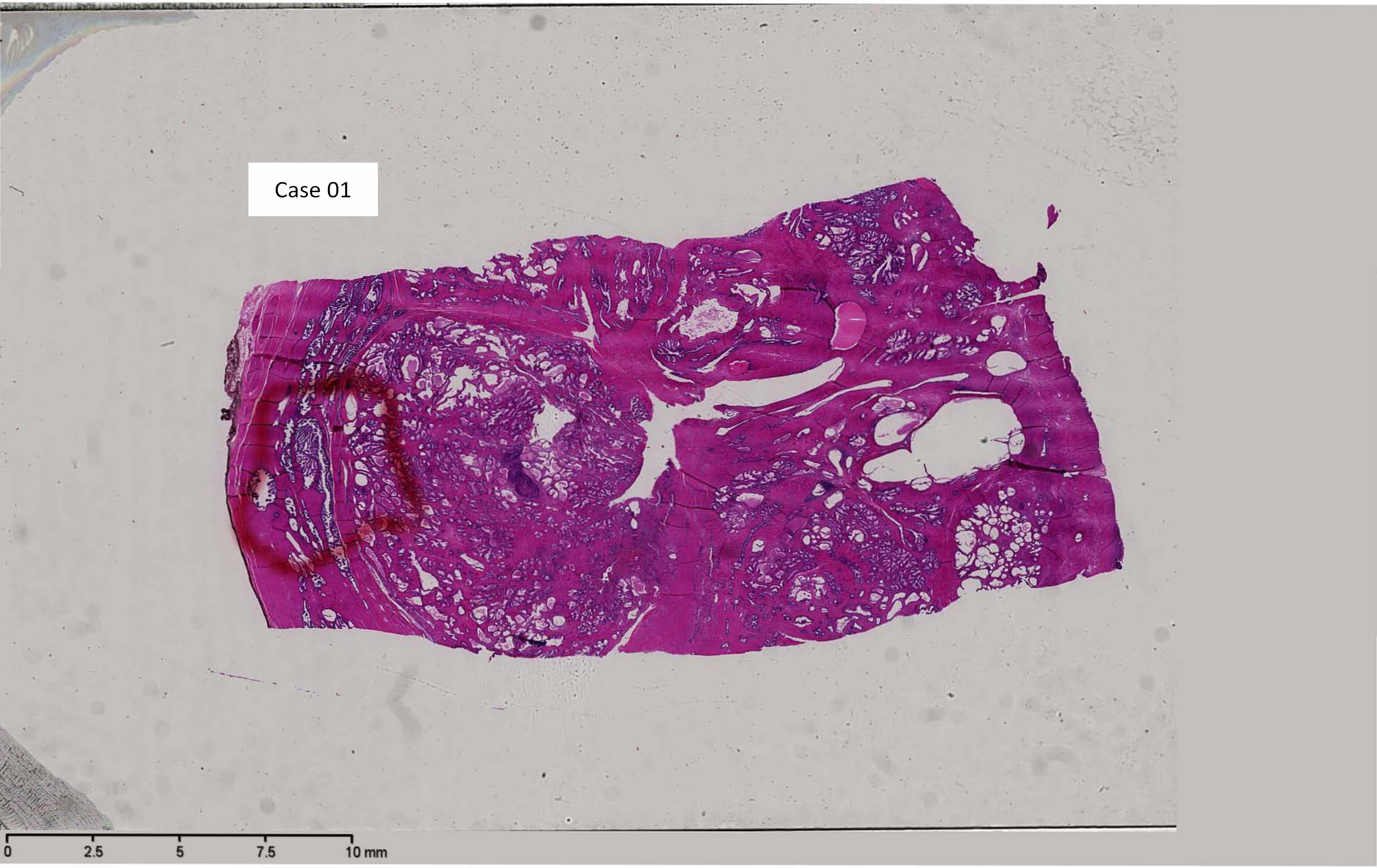

Case 02

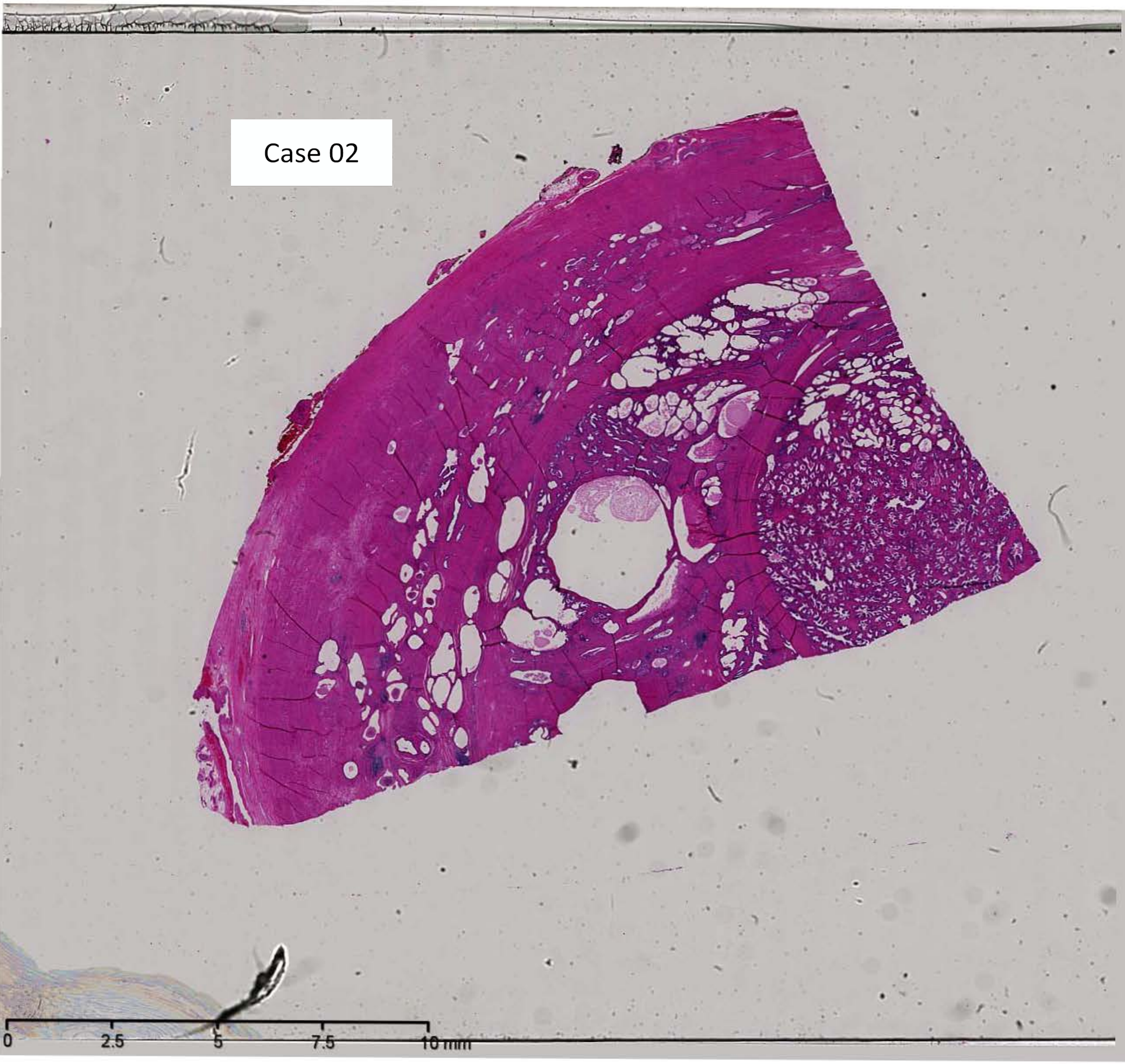

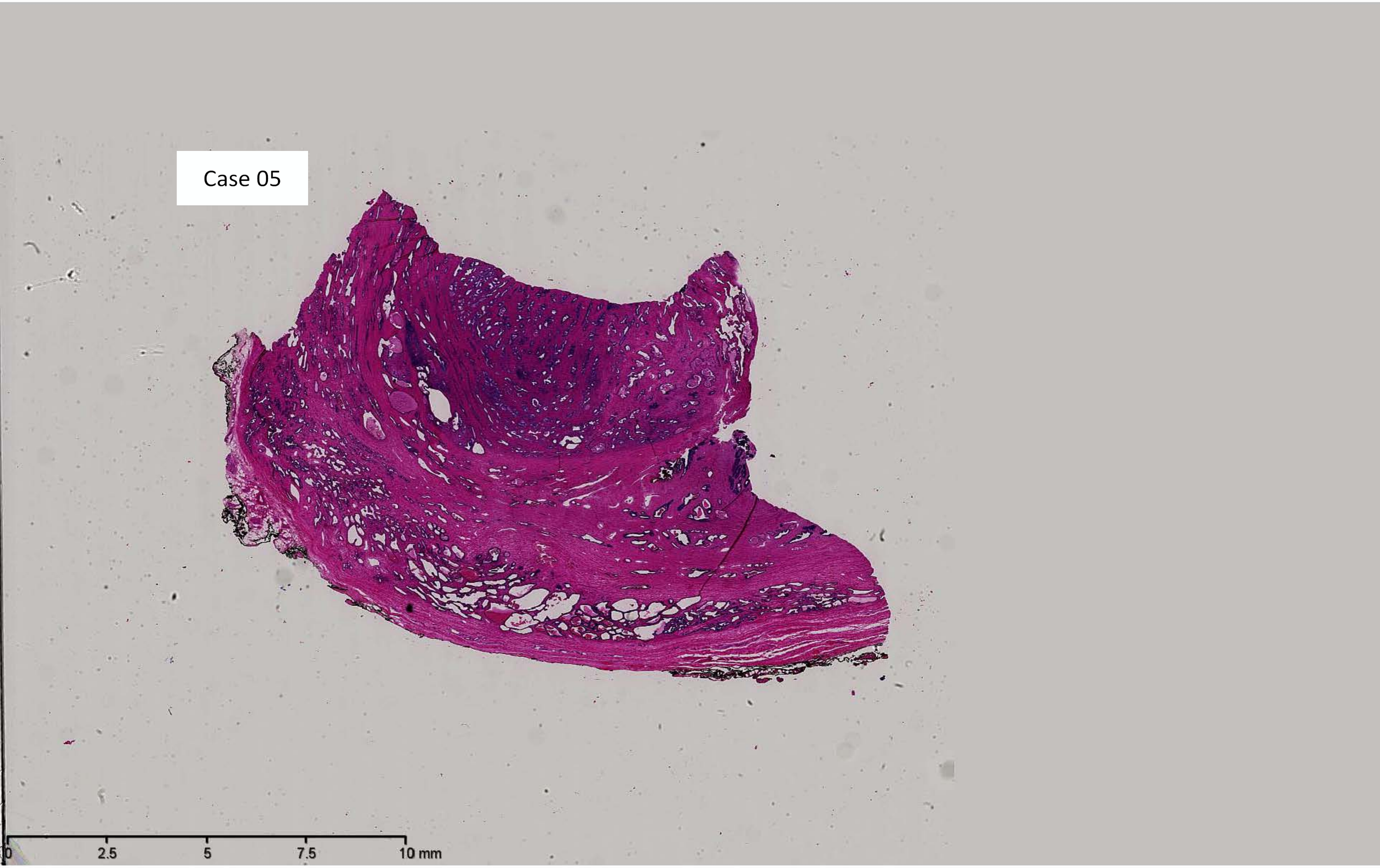
Case 05
0
2.5
5
7.5
10 mm

Case 07

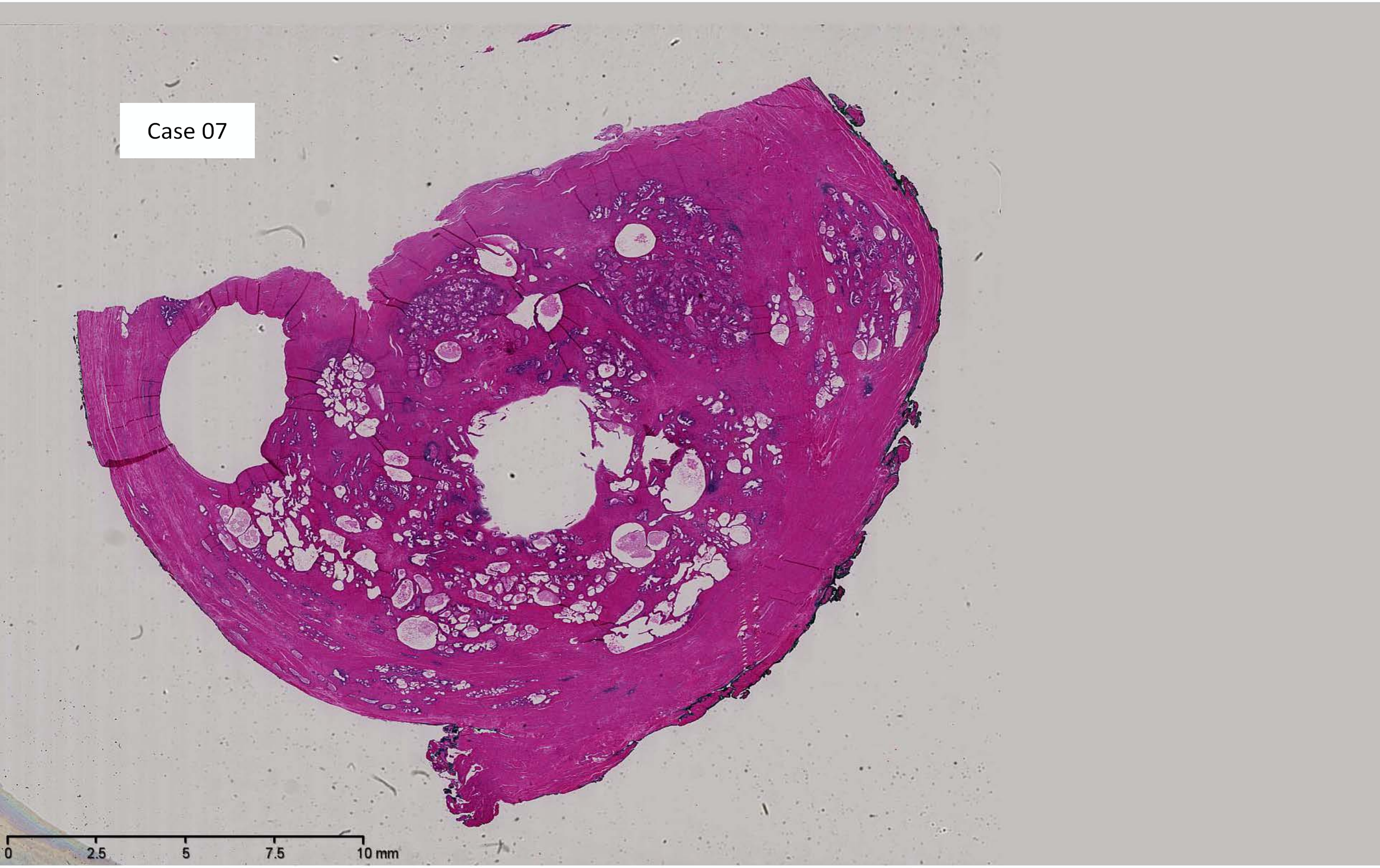

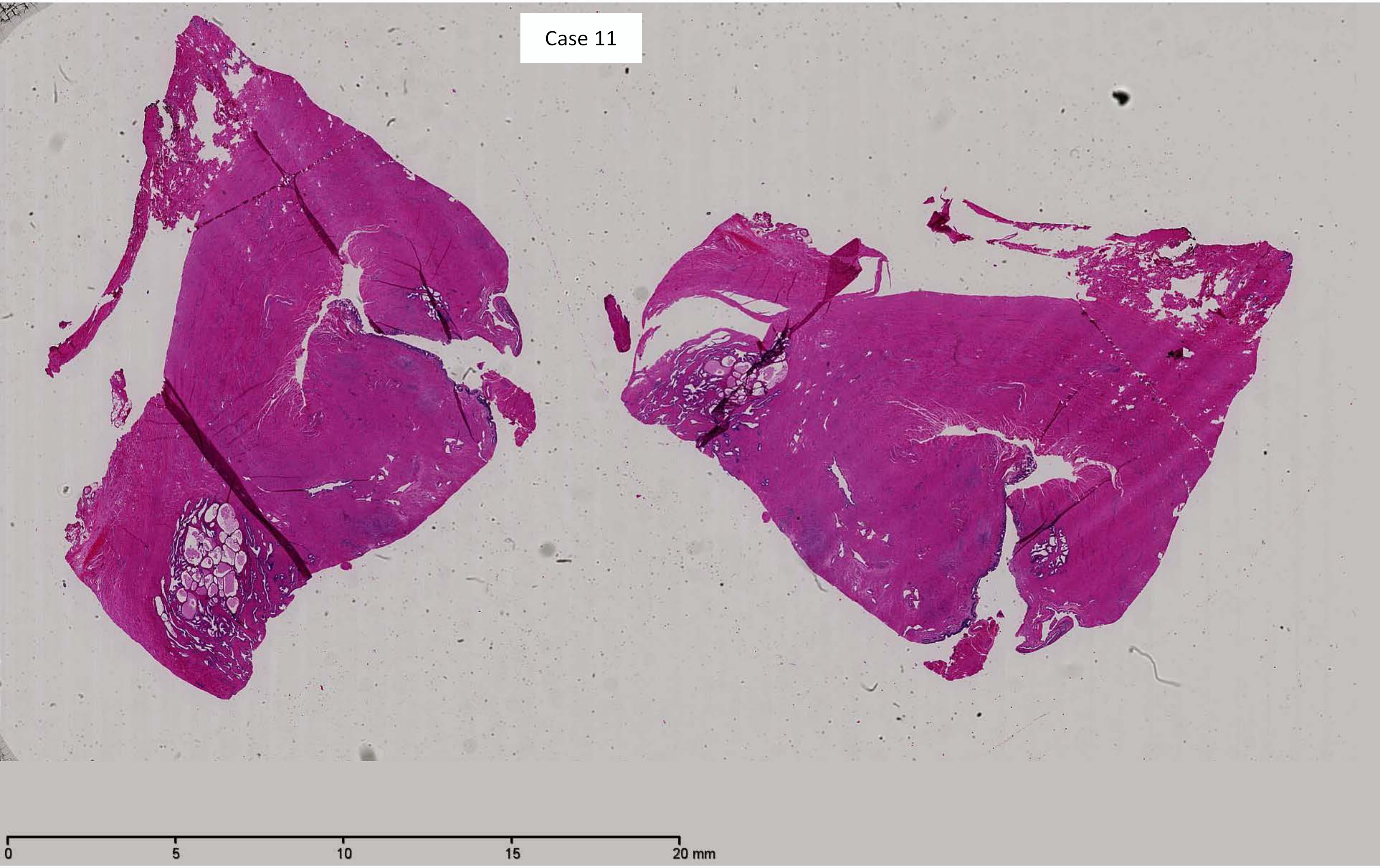
Case 11
0
5
10
15
20 mm

Case 12

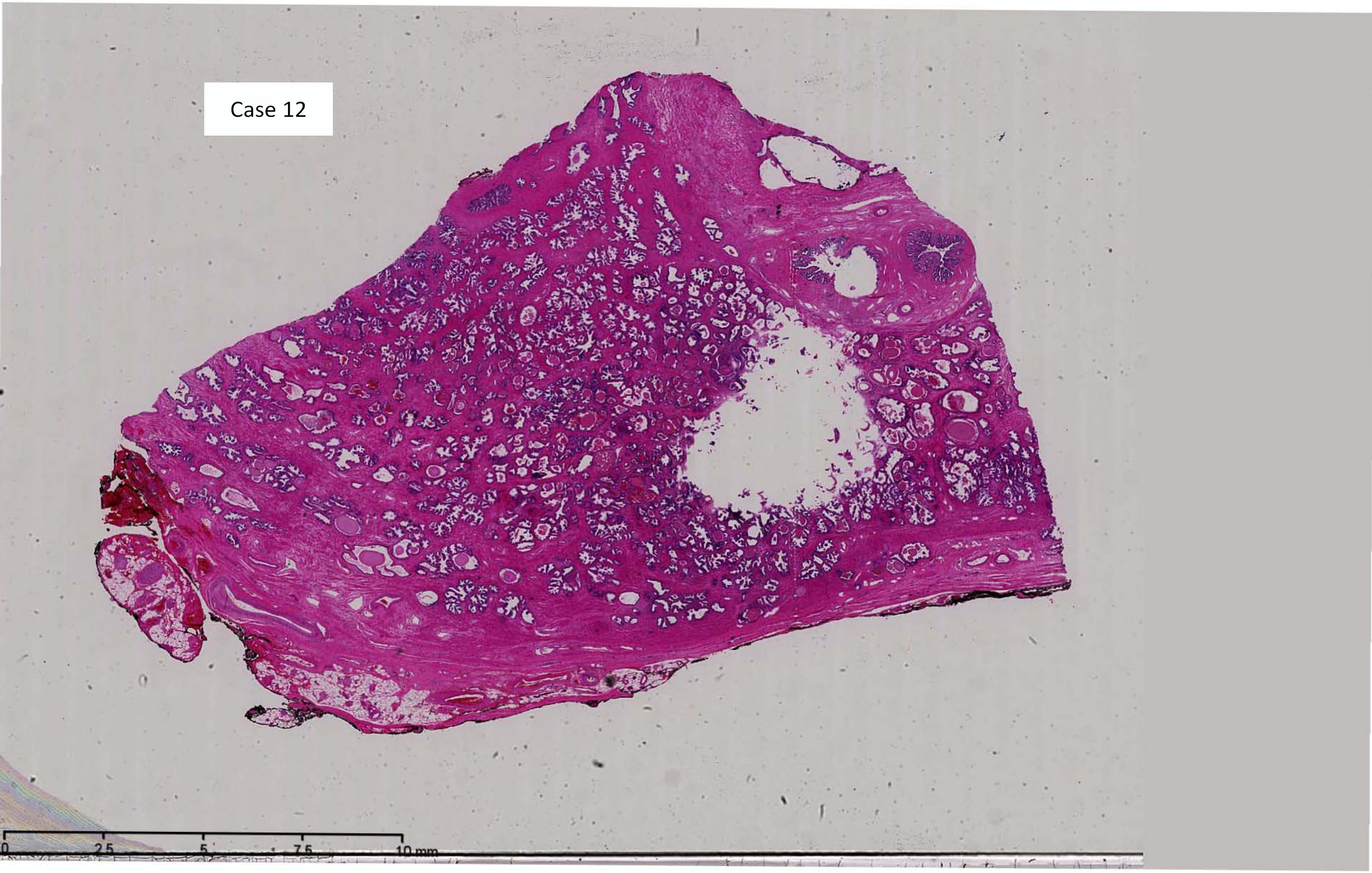

Case 13

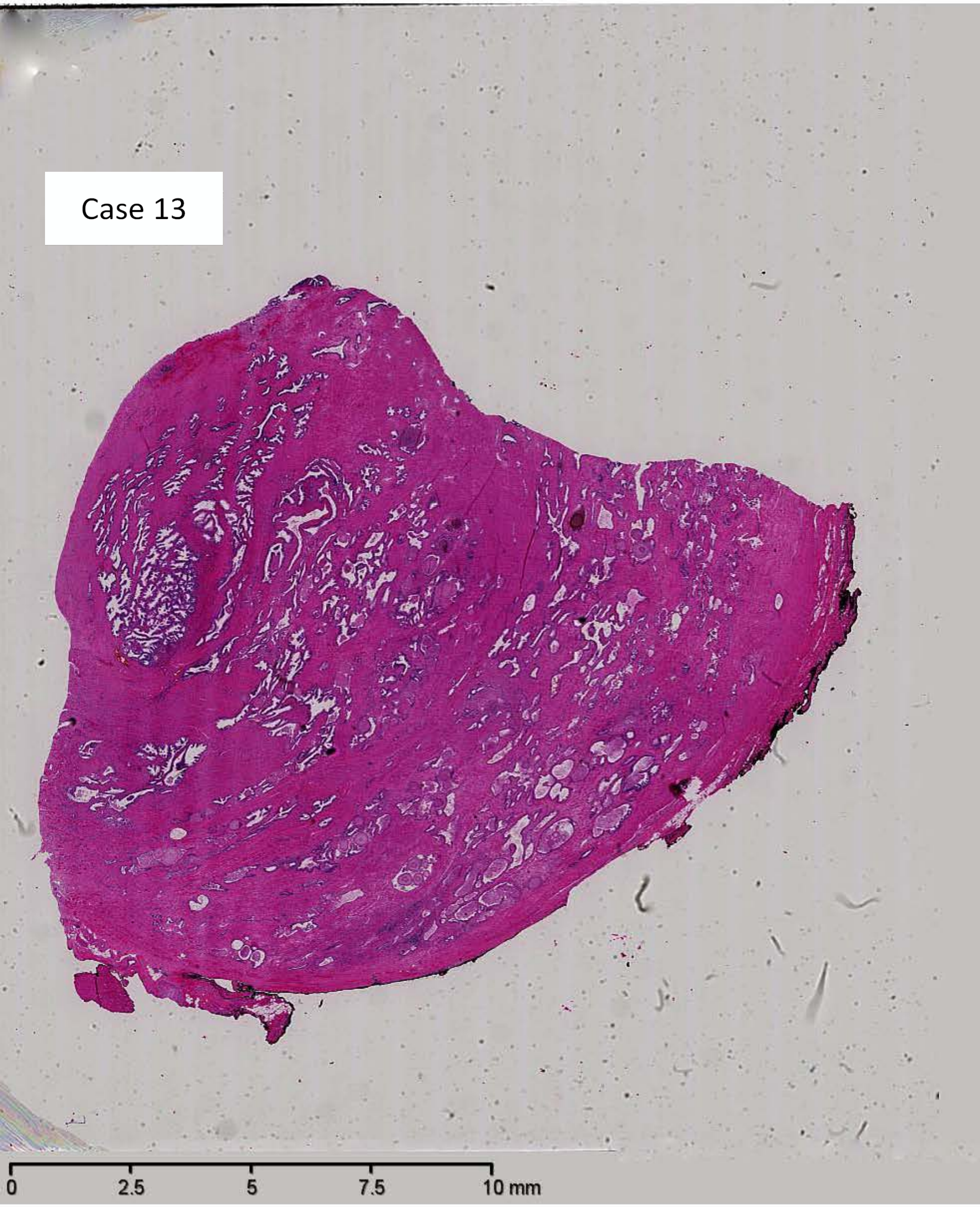

Case 14

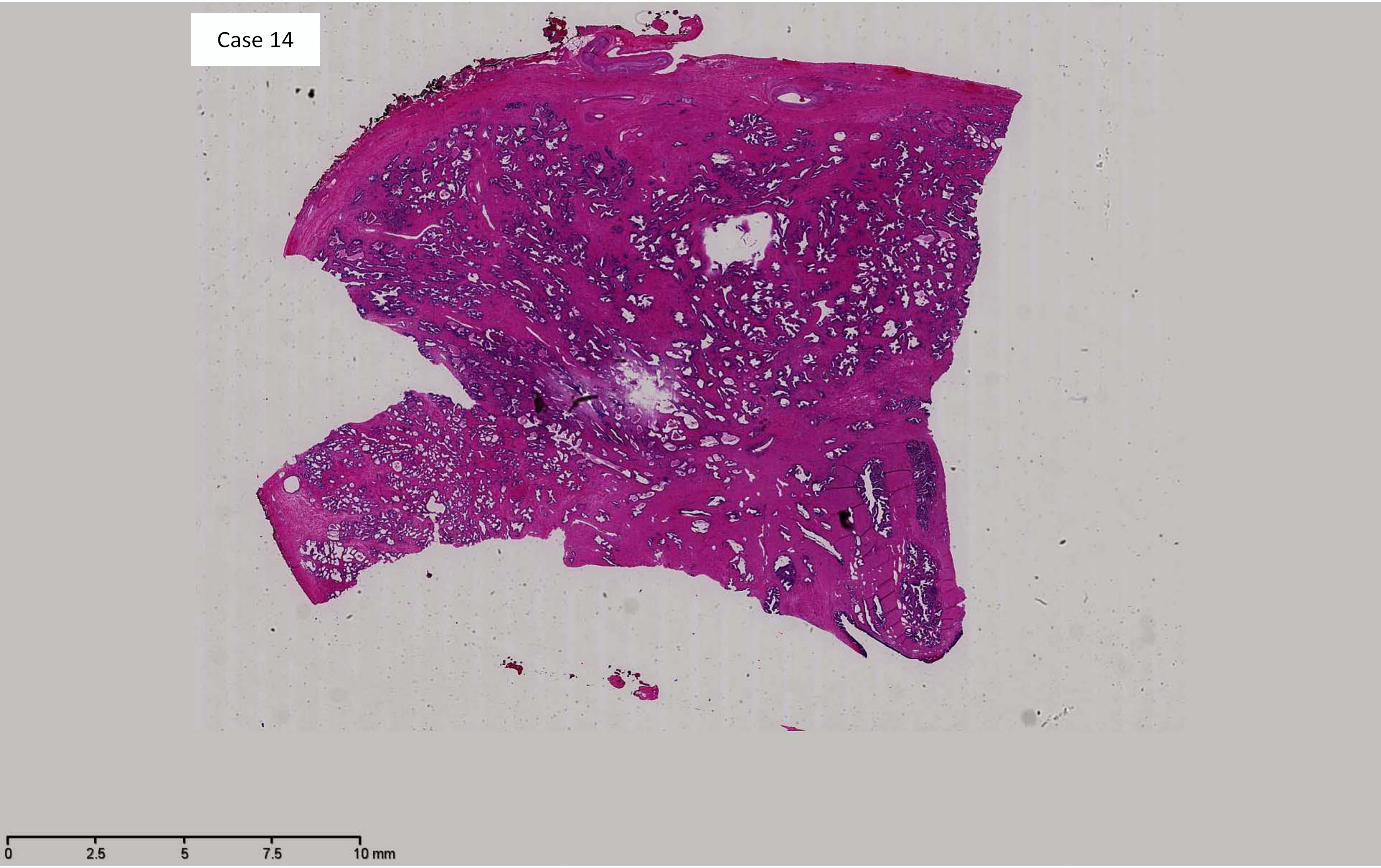

Case 15

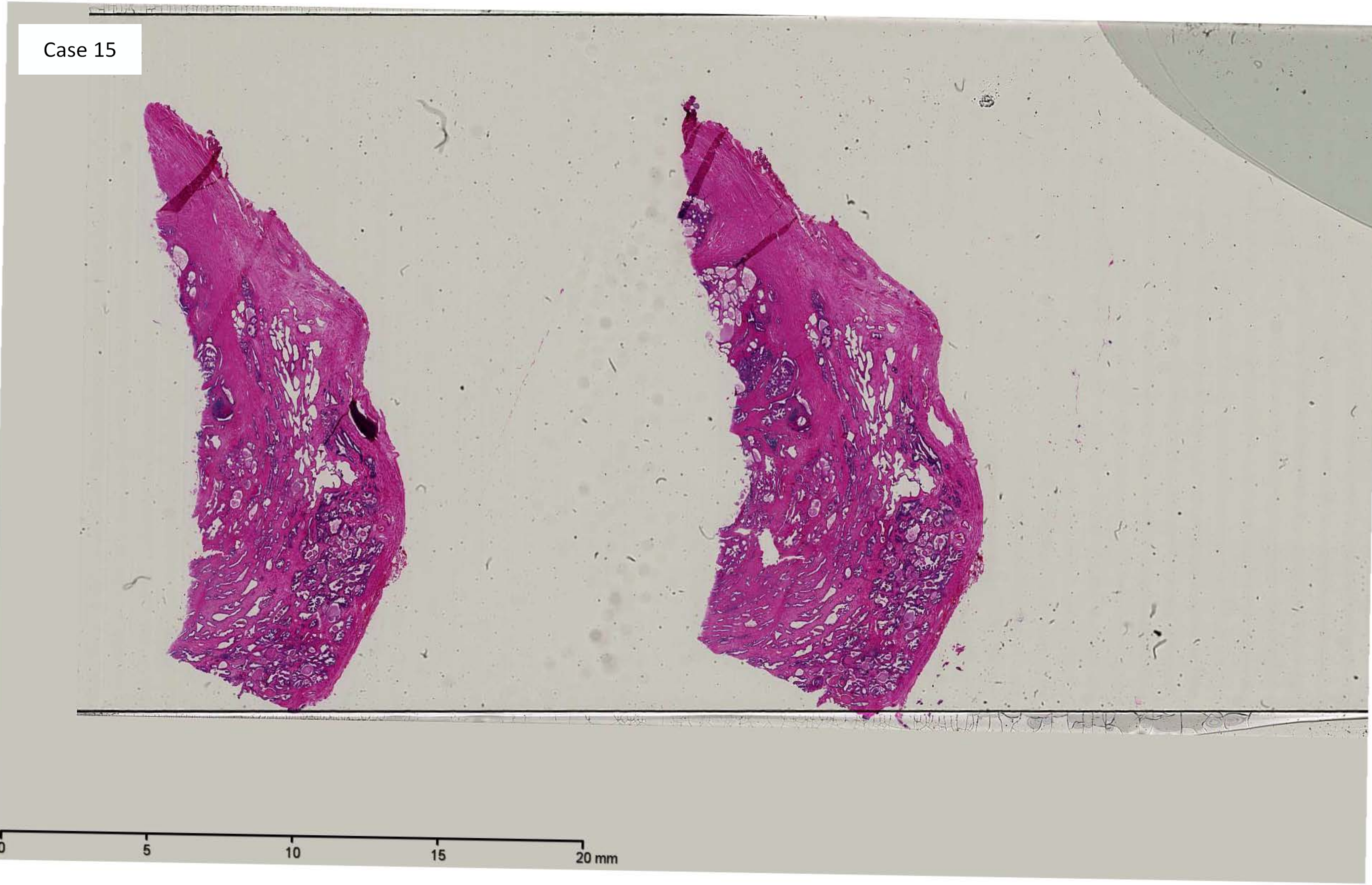

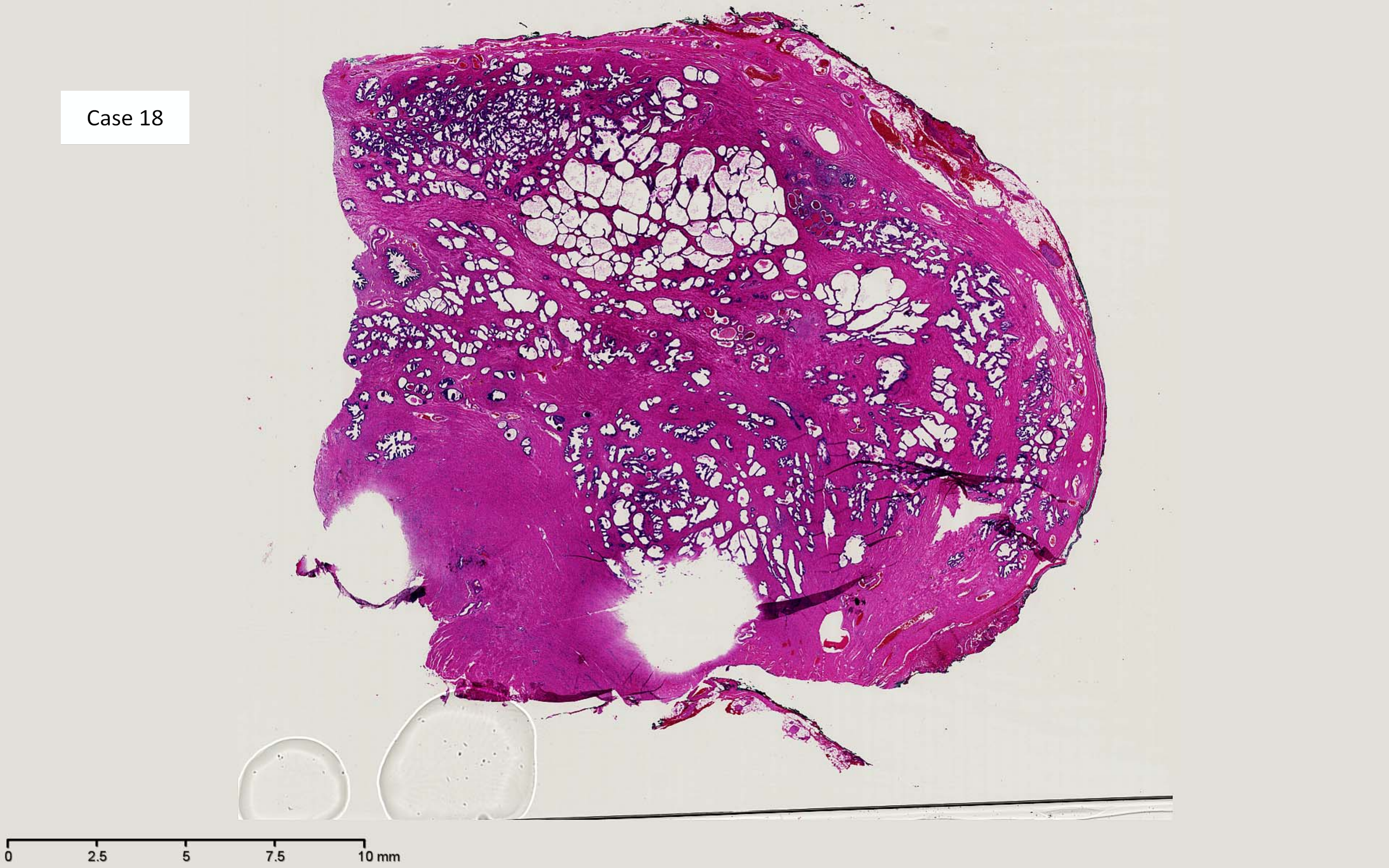
Case 18
0
2.5
5
7.5
10 mm

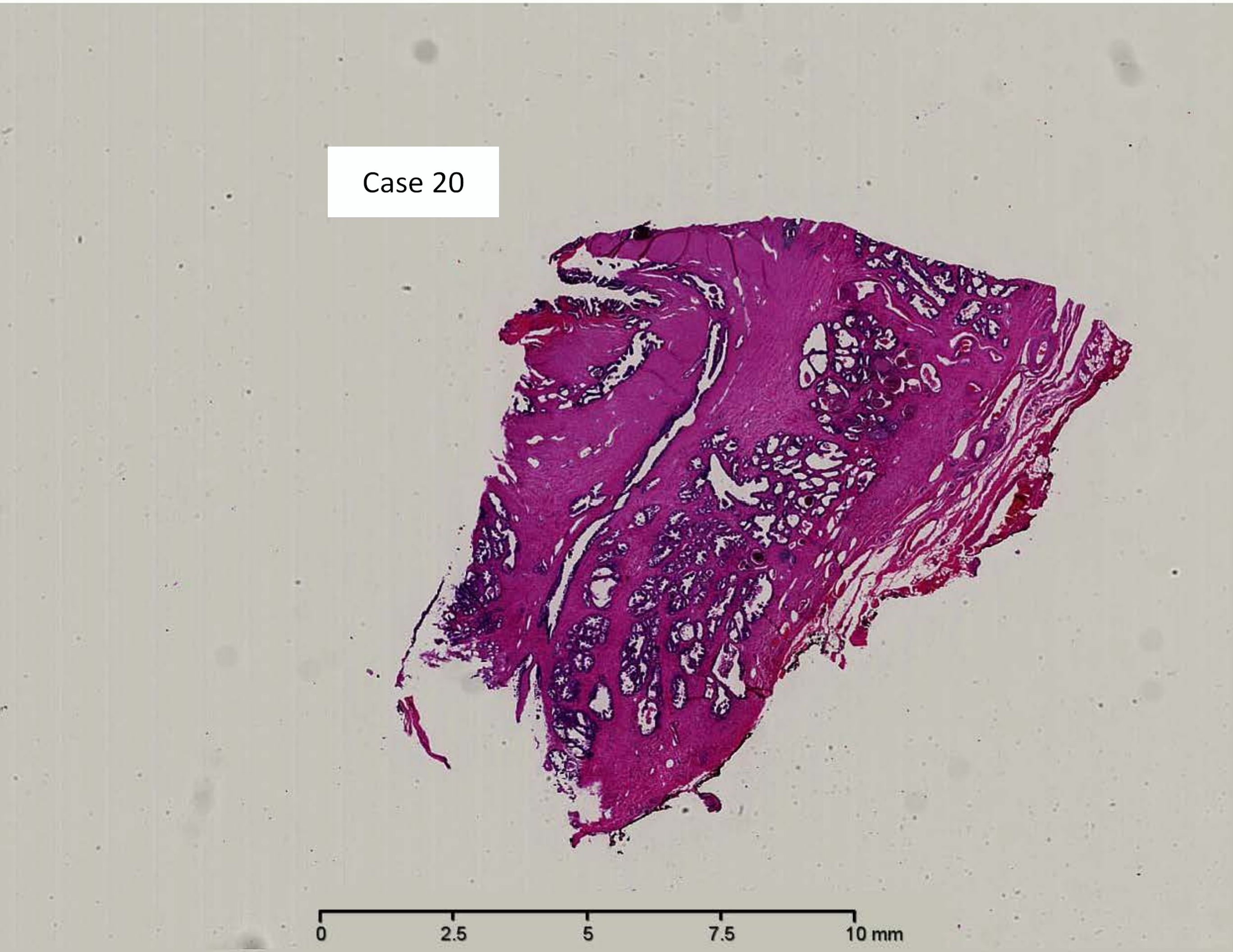
Case 20
0
2.5
5
7.5
10 mm

Case 22

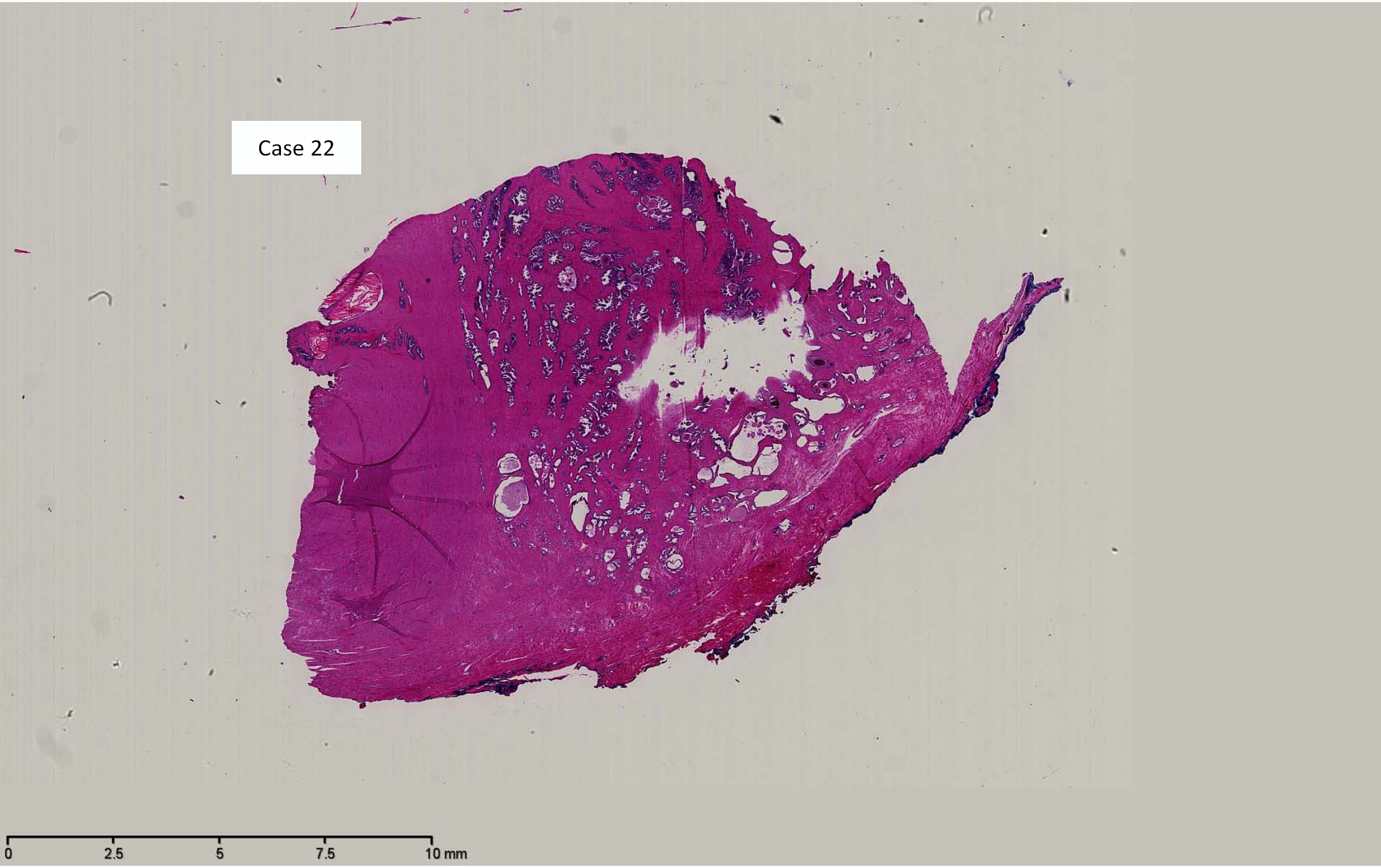

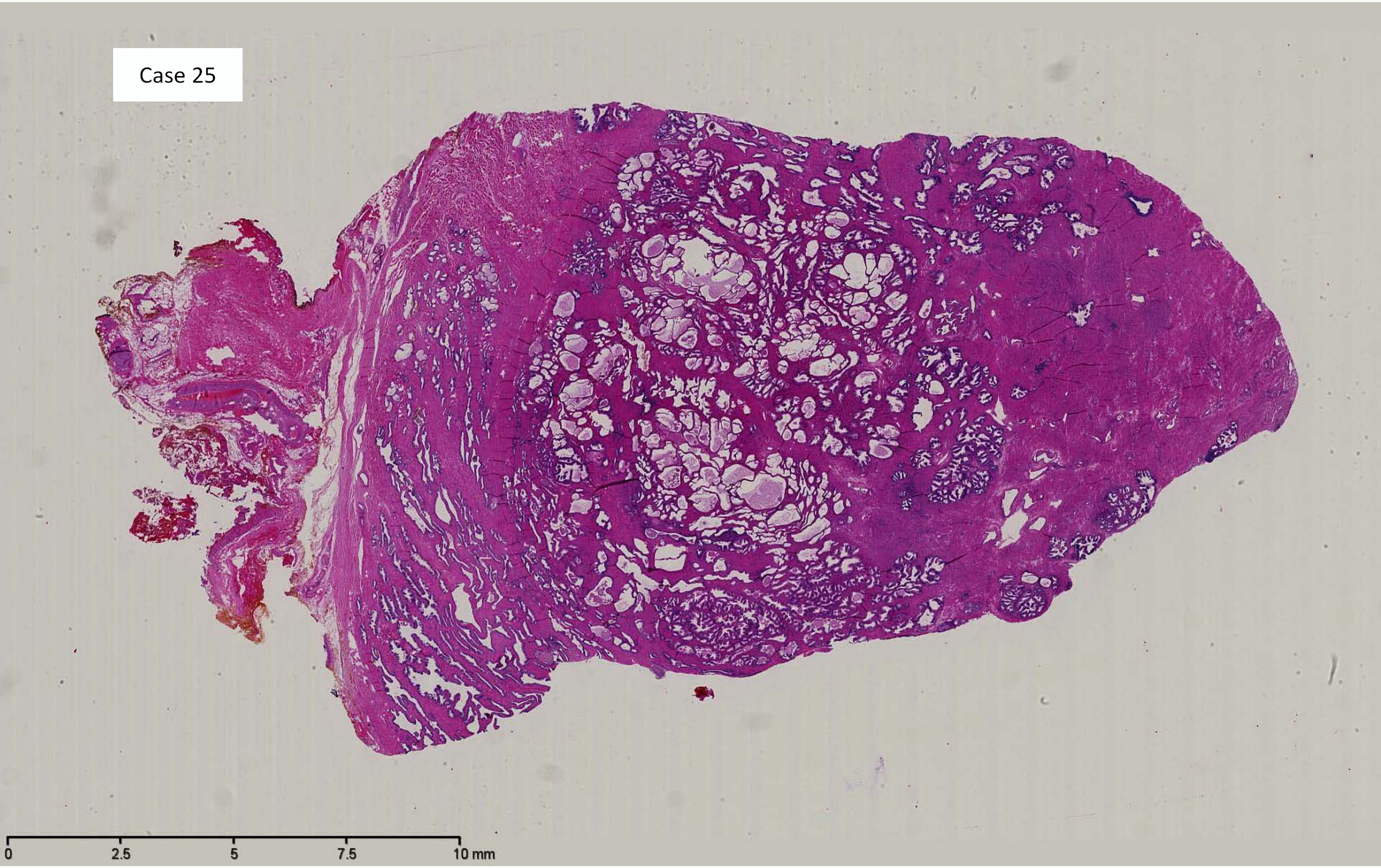
Case 25
0
2.5
5
7.5
10 mm

Case 27

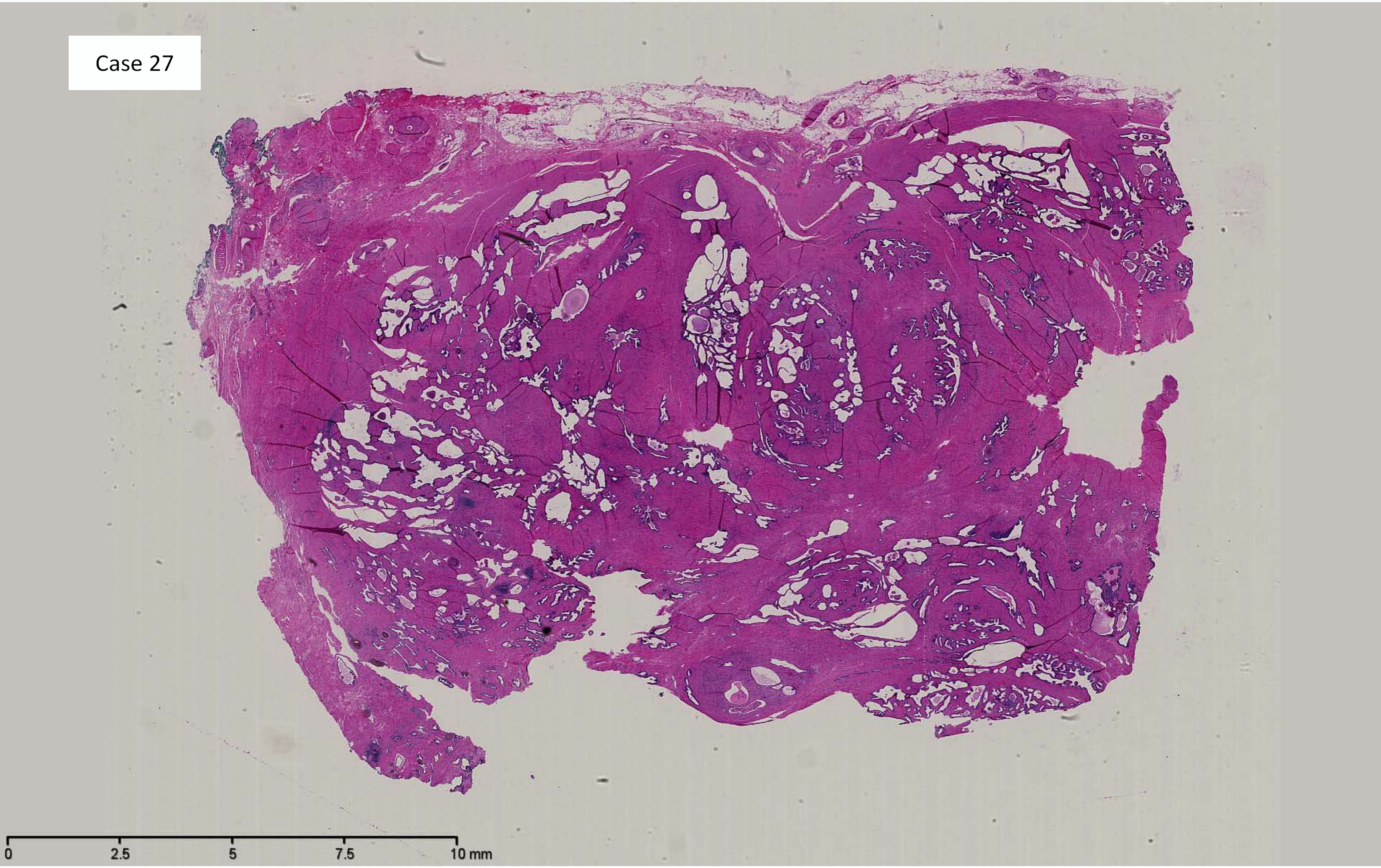

Case 29

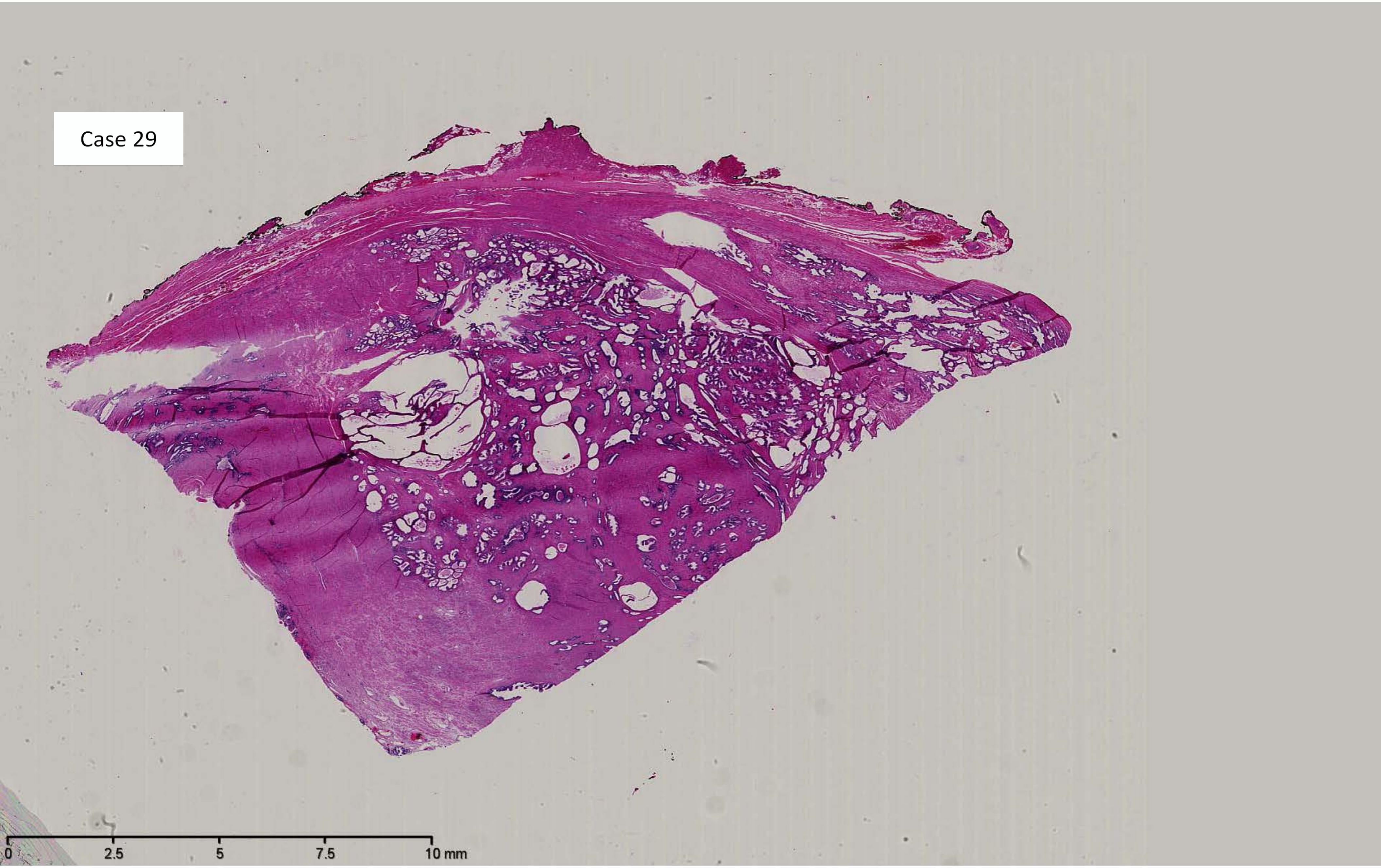

Case 32

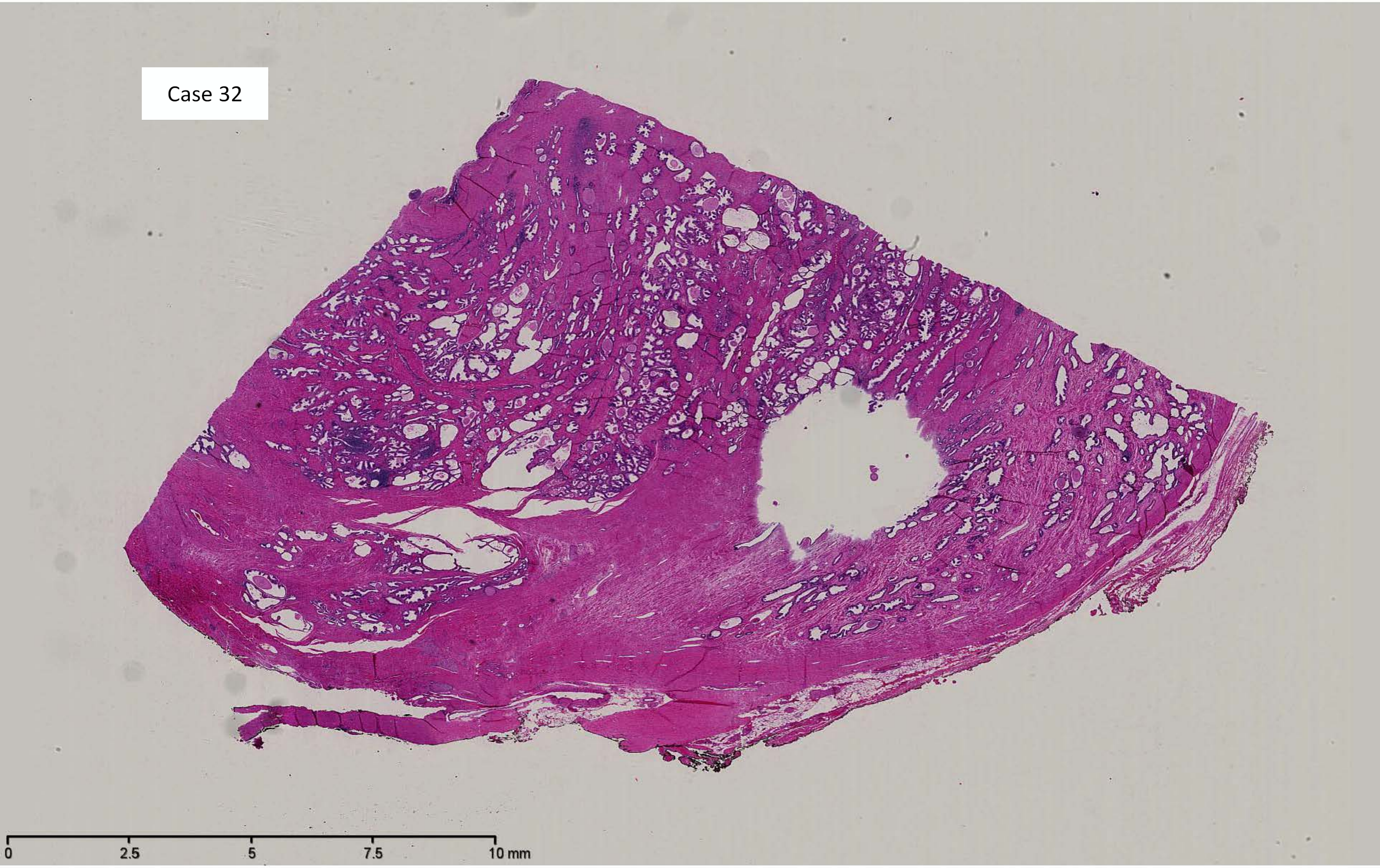

Case 33

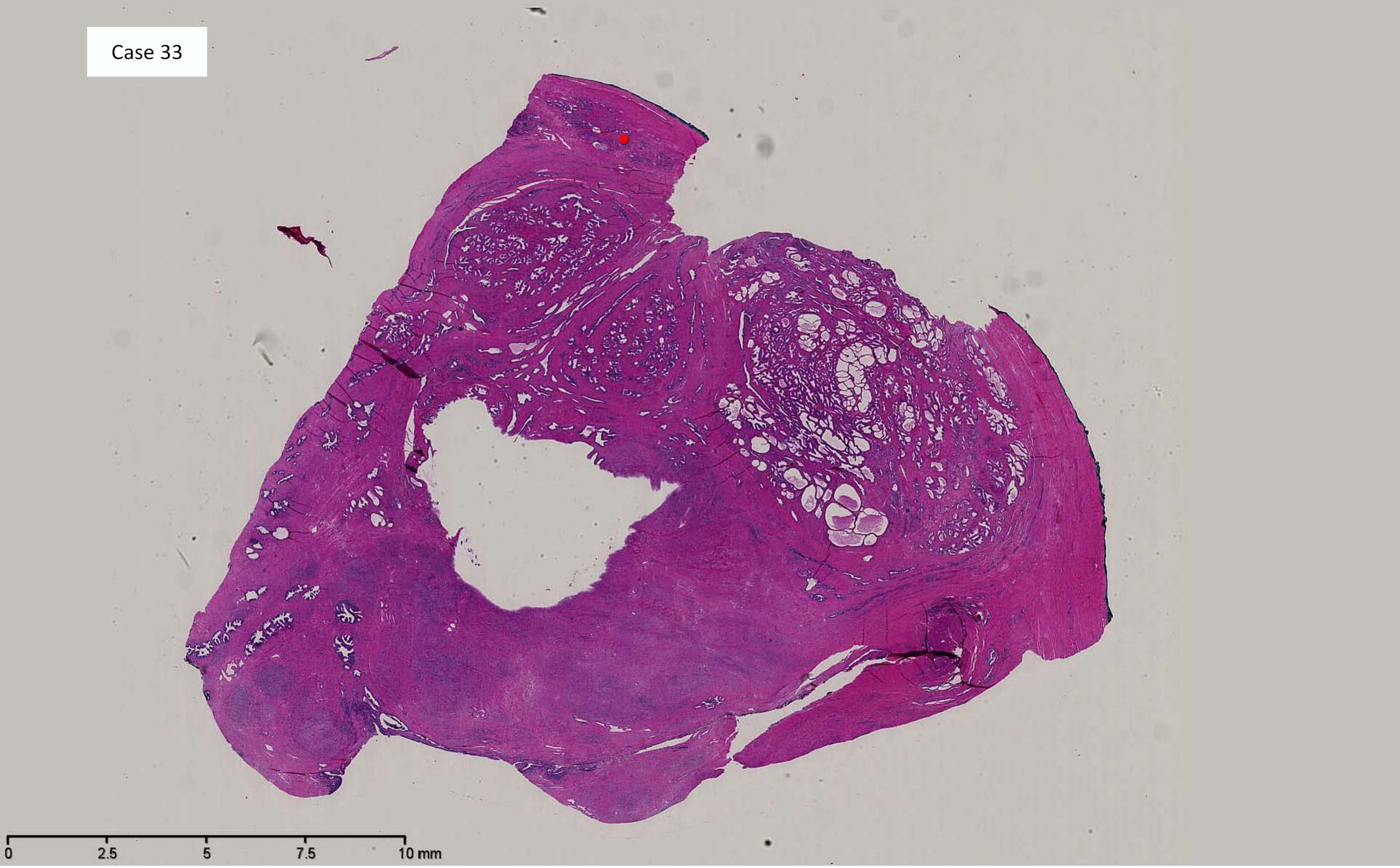

Supplement: Additional file 1 — H&E Images of the 5-μm sections of the 17 histologically benign specimens from the corresponding radical prostatectomy adenocarcinoma cases. For each case, the specimen slide used for the FISH analysis was within 10 serial sections of the H&E stained slide. [file 1471-2407-14-129-S1.pdf]
