# Supplementary material for: Identification of FISH biomarkers to detect chromosome abnormalities associated with prostate adenocarcinoma in tumour and field effect environment
Source: BMC Cancer. 2014 Feb 25;14:129. doi: 10.1186/1471-2407-14-129 (PMC4016502; doi:10.1186/1471-2407-14-129)
Supplement: Additional file 2 — t-test analyses. Table S2a.t-test of tumour ROI vs BPH. Table S2b.t-test of histologically benign ROI vs BPH. [file 1471-2407-14-129-S2.docx]

**Additional file 2**

**Table 2a.** t-test of tumour ROI vs BPH.

| **# of FISH Parameters** | **FISH Parameters** | **Tumor- Mean(Std)** | **BPH-Mean(Std)** | **p-value** | **Flag** |
| --- | --- | --- | --- | --- | --- |
| 1 | CEP10%Abnorm | 40.03(16.9) | 22.92(8.14) | <.0001 | Significant |
| 2 | CEP10%Gain | 17.21(20.49) | 2.31(4.72) | 0.0002 | Significant |
| 3 | CEP7%Abnorm | 37.83(16.99) | 19.46(6.44) | <.0001 | Significant |
| 4 | CEP7%Gain | 15.87(22.22) | 1.54(3.93) | 0.0008 | Significant |
| 5 | CEP8%Abnorm | 47.55(16.02) | 23(6.02) | <.0001 | Significant |
| 6 | CEP8%Gain | 19.97(23.73) | 1.54(2.42) | <.0001 | Significant |
| 7 | CEP8%Loss | 27.58(15.33) | 21.46(5.78) | 0.0383 | Significant |
| 8 | MYC%Gain | 25.61(29.13) | 1.85(2.71) | <.0001 | Significant |
| 9 | LPL%Abnorm | 57.45(22.86) | 22.69(8.58) | <.0001 | Significant |
| 10 | LPL%Loss | 46.95(25.91) | 21.62(8.69) | <.0001 | Significant |
| 11 | PTEN%Loss | 35.38(23.37) | 20.92(8.6) | 0.0018 | Significant |
| 12 | PTEN/CEP10%Loss | 25.54(21.41) | 7.85(4.86) | <.0001 | Significant |
| 13 | CEP7/CEP10%Gain | 19.72(13.49) | 12.15(5.36) | 0.0047 | Significant |
| 14 | LPL/CEP8%Loss | 35.89(28.69) | 8.08(4.18) | <.0001 | Significant |
| 15 | MYC/CEP8%Gain | 26.12(20.53) | 7.77(4.25) | <.0001 | Significant |
| 16 | MYC/LPL%Gain | 37.52(26.36) | 8.08(4.75) | <.0001 | Significant |

**Table 2b.** t-test of benign ROI vs BPH.

| **# of FISH Parameters** | **FISH Parameters** | **Benign- Mean(Std)** | **BPH-Mean(Std)** | **p-value** | **Flag** |
| --- | --- | --- | --- | --- | --- |
| 1 | CEP10%Abnorm | 31.41(9.91) | 22.92(8.14) | 0.0038 | Significant |
| 2 | CEP10%Gain | 5.25(4.44) | 2.31(4.72) | 0.0469 | Significant |
| 3 | CEP7%Abnorm | 29.82(8.57) | 19.46(6.44) | <.0001 | Significant |
| 4 | CEP7%Gain | 6.39(6.81) | 1.54(3.93) | 0.0139 | Significant |
| 5 | CEP8%Abnorm | 31.37(12.92) | 23(6.02) | 0.0210 | Significant |
| 6 | CEP8%Gain | 7.93(6.93) | 1.54(2.42) | 0.0017 | Significant |
| 7 | CEP8%Loss | 23.44(13) | 21.46(5.78) | 0.5611 | Not Significant |
| 8 | MYC%Gain | 9.38(8.31) | 1.85(2.71) | 0.0019 | Significant |
| 9 | LPL%Abnorm | 32.15(12.45) | 22.69(8.58) | 0.0052 | Significant |
| 10 | LPL%Loss | 24.63(11.4) | 21.62(8.69) | 0.3312 | Not Significant |
| 11 | PTEN%Loss | 34.29(16.86) | 20.92(8.6) | 0.0064 | Significant |
| 12 | PTEN/CEP10%Loss | 17.71(16.47) | 7.85(4.86) | 0.0275 | Significant |
| 13 | CEP7/CEP10%Gain | 16.65(10.26) | 12.15(5.36) | 0.1105 | Not Significant |
| 14 | LPL/CEP8%Loss | 11.73(9.45) | 8.08(4.18) | 0.1487 | Not Significant |
| 15 | MYC/CEP8%Gain | 13.44(12.84) | 7.77(4.25) | 0.0951 | Not Significant |
| 16 | MYC/LPL%Gain | 13.97(11.64) | 8.08(4.75) | 0.0618 | Not Significant |
