# Supplementary material for: Identification of FISH biomarkers to detect chromosome abnormalities associated with prostate adenocarcinoma in tumour and field effect environment
Source: BMC Cancer. 2014 Feb 25;14:129. doi: 10.1186/1471-2407-14-129 (PMC4016502; doi:10.1186/1471-2407-14-129)
Supplement: Additional file 3 — The pattern of FISH abnormalities presents in the 33 tumour ROIs and the 17 available corresponding histologically benign ROIs. [file 1471-2407-14-129-S3.docx]

**Additional file 3**.

| **Case  ID** | **Tumor Section** | | | | **Histologically Benign Section** | | | |
| --- | --- | --- | --- | --- | --- | --- | --- | --- |
|  | **PTEN** | **CEP7** | **MYC** | **CEP8** | **PTEN** | **CEP7** | **MYC** | **CEP8** |
| 01 |  |  |  |  |  |  |  |  |
| 02 |  |  |  |  |  |  |  |  |
| 03 |  |  |  |  |  |  |  |  |
| 04 |  |  |  |  |  |  |  |  |
| 05 |  |  |  |  |  |  |  |  |
| 06 |  |  |  |  |  |  |  |  |
| 07 |  |  |  |  |  |  |  |  |
| 08 |  |  |  |  |  |  |  |  |
| 09 |  |  |  |  |  |  |  |  |
| 10 |  |  |  |  |  |  |  |  |
| 11 |  |  |  |  |  |  |  |  |
| 12 |  |  |  |  |  |  |  |  |
| 13 |  |  |  |  |  |  |  |  |
| 14 |  |  |  |  |  |  |  |  |
| 15 |  |  |  |  |  |  |  |  |
| 16 |  |  |  |  |  |  |  |  |
| 17 |  |  |  |  |  |  |  |  |
| 18 |  |  |  |  |  |  |  |  |
| 19 |  |  |  |  |  |  |  |  |
| 20 |  |  |  |  |  |  |  |  |
| 21 |  |  |  |  |  |  |  |  |
| 22 |  |  |  |  |  |  |  |  |
| 23 |  |  |  |  |  |  |  |  |
| 24 |  |  |  |  |  |  |  |  |
| 25 |  |  |  |  |  |  |  |  |
| 26 |  |  |  |  |  |  |  |  |
| 27 |  |  |  |  |  |  |  |  |
| 28 |  |  |  |  |  |  |  |  |
| 29 |  |  |  |  |  |  |  |  |
| 30 |  |  |  |  |  |  |  |  |
| 31 |  |  |  |  |  |  |  |  |
| 32 |  |  |  |  |  |  |  |  |
| 33 |  |  |  |  |  |  |  |  |

FISH Positive

FISH Negative

Specimen not available
